# Transforming Sleep Monitoring: Review of Wearable and Remote Devices Advancing Home Polysomnography and Their Role in Predicting Neurological Disorders

**DOI:** 10.3390/bios15020117

**Published:** 2025-02-17

**Authors:** Diana Vitazkova, Helena Kosnacova, Daniela Turonova, Erik Foltan, Martin Jagelka, Martin Berki, Michal Micjan, Ondrej Kokavec, Filip Gerhat, Erik Vavrinsky

**Affiliations:** 1 Institute of Electronics and Photonics, Faculty of Electrical Engineering and Information Technology, Slovak University of Technology, Ilkovicova 3, 81219 Bratislava, Slovakia; helena.kosnacova@stuba.sk (H.K.); erik.foltan@stuba.sk (E.F.); martin.jagelka@stuba.sk (M.J.); martin.berki@stuba.sk (M.B.); michal.micjan@stuba.sk (M.M.); ondrej.kokavec@stuba.sk (O.K.); filip.gerhat@stuba.sk (F.G.); 2 Department of Psychology, Faculty of Arts, Comenius University, Gondova 2, 81102 Bratislava, Slovakia; daniela.moskalova@uniba.sk

**Keywords:** human sleep monitoring, polysomnography, home environment, respiration, photoplethysmography, neurodegenerative diseases

## Abstract

This paper explores the progressive era of sleep monitoring, focusing on wearable and remote devices contributing to advances in the concept of home polysomnography. We begin by exploring the basic physiology of sleep, establishing a theoretical basis for understanding sleep stages and associated changes in physiological variables. The review then moves on to an analysis of specific cutting-edge devices and technologies, with an emphasis on their practical applications, user comfort, and accuracy. Attention is also given to the ability of these devices to predict neurological disorders, particularly Alzheimer’s and Parkinson’s disease. The paper highlights the integration of hardware innovations, targeted sleep parameters, and partially advanced algorithms, illustrating how these elements converge to provide reliable sleep health information. By bridging the gap between clinical diagnosis and real-world applicability, this review aims to elucidate the role of modern sleep monitoring tools in improving personalised healthcare and proactive disease management.

## 1. Introduction

Sleep is an integral part of human life, representing an essential physiological process necessary for physical recovery, emotional balance, and the maintenance of cognitive functions [1].

The study of sleep and related disorders is the subject of a specialised medical field called sleep medicine. Sleep medicine is considered a young field whose emergence is closely related to progress in electrophysiological methods that allow monitoring of various parameters suitable for sleep evaluation [2,3]. Global sleep disorders, including chronic sleep deprivation, insomnia, obstructive sleep apnoea (OSA), and circadian rhythm disorders, have become a global epidemic that threatens the health and well-being of a large portion of the population [4]. These conditions are often underdiagnosed, poorly managed, and untreated. Chronic insomnia, which affects 30% of adults worldwide, is the most widespread sleep disorder [5]. As a result of poor sleep quality, cognitive functions, memory problems, and the effectiveness of the immune system are impaired, which can leave individuals vulnerable to infections. Chronic insufficient sleep is also linked to a higher risk of mortality. Recent studies suggest that extending the length of nightly sleep for individuals who regularly suffer from insufficient sleep may provide notable health benefits [6].

**Figure 1:**
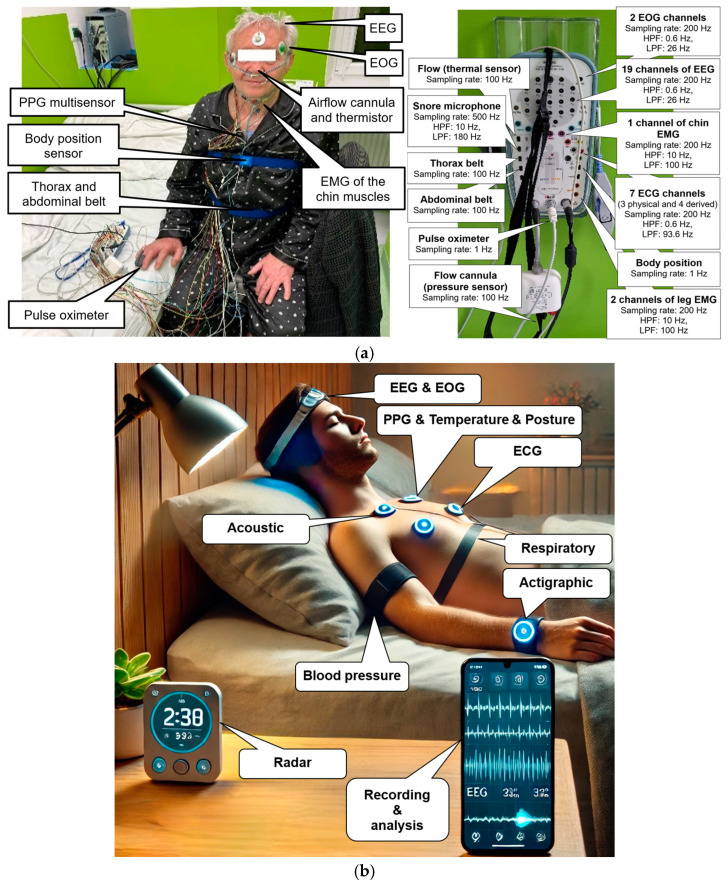
Monitoring in sleep laboratory vs. wearable sleep technologies: (**a**) Standard PSG setup, including electrode montage and a close-up of the Alice 6 LDxS Headbox (Philips Respironics) with sampling rates, low pass filter (LPF) and high pass filter values; (**b**) schematic representation of a wearable sleep monitoring concept, integrating multiple sensor technologies: an EEG and EOG headband, an advanced respiratory belt, a single-channel ECG sensor, a multi-sensor for PPG, posture, and temperature, an acoustic device for breathing sound analysis, an actigraphic device, a blood pressure monitor, a radar sensor embedded in an alarm clock, and a mobile phone serving as a recording, database, and analysis hub (produced with the help of ChatGPT 4o mini).

Diagnosis of sleep disorders is traditionally carried out in specialised sleep laboratories, which are usually part of pulmonary, psychiatric, neurological, or paediatric clinics. The standard sleep examination performed in clinical facilities is known as polysomnography (PSG) (Figure 1a), which is an overnight diagnostic procedure with a robust system [7,8,9] and specialised software that allows the recording and analysis of biosignals [10]. For comprehensive sleep assessments, PSG remains the gold standard among all sleep examinations. The advantage of PSG lies in its precise and comprehensive measurement of physiological parameters, which offers valuable insights into the overall sleep patterns and issues of an individual. During overnight video-polysomnography, patients are monitored under medical supervision while numerous sensors are attached to the body. The PSG configuration includes simultaneous recording of electrocardiography (ECG), electrooculography (EOG), electroencephalography (EEG), electromyography (EMG), respiration, abdominal and thoracic respiratory effort, snoring, heart rate (HR), blood oxygen saturation (SpO_2_), body position, and all body movements. The examination is monitored using an infrared (IR) camera to capture pathological movements during sleep [10,11].

This process requires specialised expertise in polysomnographic assembly and subsequent biosignals processing, and it is both time-consuming and expensive. The sleep quality of patients undergoing polysomnography is often disrupted by the discomfort caused by the multitude of wires and sensors attached to the body that restricts natural movement during sleep. The number of individuals with sleep disorders is growing, which significantly affects waiting times for PSG examinations. This increase is closely associated to the rising incidence of OSA, a disease for which obesity is a major risk factor. Given that obesity is now considered a global health crisis, the challenges related to addressing these issues are increasingly urgent [12]. In this context, another challenge arises in the availability of diagnostic tests. For example, waiting times for PSG in our country, Slovakia, typically range from six months to 1.5 years. This situation may lead to delayed diagnosis and subsequent treatment of OSA patients, which may have negative health consequences.

The increasing prevalence of sleep disorders, combined with the long waiting times and high costs associated with traditional sleep diagnostics, has spurred interest in the development of wearable sleep monitoring devices. Home PSG (Figure 1b) represents a promising alternative to traditional diagnostic methods, because it enables non-invasive, more comfortable, less intrusive, and long-term sleep monitoring in the patient’s natural environment. Reduced costs are also a significant benefit, as the application of wearable devices is simpler and does not require medical supervision. Home PSG is transforming sleep monitoring by bridging the gap between clinical diagnosis and patient convenience. This tool refines detailed assessments of sleep architecture and temporal changes, enabling early detection, more accurate risk assessment, and targeted disease management that reduces the pressure on sleep labs and healthcare professionals. Thanks to advances in new technologies, wireless communication, and artificial intelligence, wearable devices are becoming increasingly sophisticated and widely available, enabling detailed assessment of temporal changes in sleep architecture, as well as obtaining earlier detection and more accurate and efficient diagnostics, which may lead to a revolution in the field of sleep medicine. Studies have shown that home PSG can provide reliable and high-quality results comparable to those obtained under laboratory settings [13,14,15].

The importance of home polysomnographic monitoring is evident from its potential to facilitate early detection and diagnosis of neurological disorders, in which subtle sleep disturbances often serve as critical early indicators. Neurodegenerative pathologies such as Alzheimer’s disease (AD) and Parkinson’s disease (PD) represent another increasing burden on the healthcare system, and it is therefore appropriate to look for early symptoms to help treat these diseases before the onset of their clinical symptoms. Many studies indicate cognitive, behavioural, sensory, and motor changes preceding clinical manifestations of these diseases. Therefore, monitoring using mobile and wearable technologies in the home environment again appears to be ideal [16]. Sleep abnormalities often occur in neurodegenerative diseases. However, whether these constitute a suitable parameter for early diagnosis or whether they appear later in the course of the disease is still a subject of investigation. Sleep and neurodegeneration have a bidirectional relationship, because sleep-regulating centres are affected by diseases, and sleep is also associated with the acceleration and worsening of diseases, because proteins are not removed during sleep and oxidative stress is increased [17]. AD and PD are progressive and the two most common neurodegenerative disorders. As the number of elderly people in the population increases, so does the number of cases of these diseases, the prevalence of which correlates with older age. AD and PD currently have no effective treatments; available therapies only alleviate symptoms and slow their progression. They are usually diagnosed only after the onset of these symptoms, and early diagnosis is essential for initiating treatment as soon as possible. Patients have impaired thinking and motor skills, but sleep disorders such as insomnia, hypersomnia, and excessive daytime sleepiness have also been found in them. This may stem from damage to the centres in the brain that control sleep cycles and where neurotoxic forms of amyloid β-peptide, tau, and α-synuclein accumulate in these diseases [18,19]. For example, treatment of sleep-controlling signalling pathways in animal models has been shown to slow the progression of AD and PD. In any case, sleep disturbances are associated with these diseases and may predict disease progression [20,21,22,23]. Studies suggest that early detection of certain sleep patterns may help identify individuals at higher risk of dementia [17,18].

The increasing focus on sleep disorders as early markers of neurological disorders underscores the urgent need for accessible and reliable diagnostic tools. This review explores the potential of wearable and remote devices to advance home polysomnography, paving the way for early detection of neurodegenerative disorders based on home monitoring and improving patient outcomes by initiating treatment as early as possible.

As can be seen from the introduction, the motivation to write this review is high. However, deciding which studies and devices to include is more difficult. Essentially, the analysis of any physiological variable during sleep can qualify as sleep monitoring. Therefore, we focused on selecting wearable and remote devices that offer added value, have unique features, and ultimately contribute to the development of home PSG. The article is structured into two main chapters. Section 2, “Physiology of Sleep”, provides an overview of sleep stages, physiological changes during the night, and how these changes can be affected by neurological diseases. We believe that this theoretical foundation is essential, as any progressive sleep monitoring device must build on top of it. Section 3, “Current State of Technological Evolution”, focuses on the current state of sleep monitoring research and highlights the most promising devices. It starts with basic systems, categorised by the main physiological parameter. In the second subsection, we shift our focus to advanced multi-sensors, quasi-“PSG” products. Each subsection is supplemented with summary technical tables, and the chapter ends with a section on how some of these devices are applied in the research and prediction of neurological disorders. The article finally concludes with a short discussion that touches on future directions of our research and interesting aspects that could not be discussed in detail in the main text, followed by an overall conclusion.

## 2. Physiology of Sleep

### 2.1. Sleep Phases

Sleep is divided into two phases: non-rapid eye movement (NREM) and rapid eye movement (REM). Individual sleep phases can be mostly distinguished through different patterns of brain activity, eye movements, and chin muscle tone. NREM sleep comprises most of sleep and consists of three stages: falling asleep (NREM 1), which is the state between wakefulness and light sleep, light sleep (NREM 2), and deep sleep (NREM 3). These stages are characterised by synchronised electroencephalogram (EEG) activity with specific markers, such as K-complexes and/or sleep spindles. During NREM sleep, activity of the brain and muscles is decreased, allowing the body to relax and recover. In contrast, REM sleep is identified by typical rapid eye movements, intense brain activity, a desynchronised EEG, frequent dreaming, and muscle atonia, except for the muscles controlling breathing and eye movements [24].

In healthy adults, the NREM and REM sleep phases alternate, repeating four to six times throughout the night during uninterrupted sleep. A complete sleep cycle for adults lasts approximately 90 to 110 min. About 75% of sleep time is spent in the NREM phase, mainly in light sleep. The first REM sleep phase is the shortest and occurs before midnight. It gradually lengthens as sleep continues, and the longest REM phase occurs in the early morning. Deep sleep shortens as the night progresses [25]. The recommended sleep length for adult people is 7 to 9 h [26]. The duration of each sleep stage changes with age. Unlike in children and adults, sleep cycles in newborns are shorter. Their sleep consists of quiet (like NREM), active (like REM), and indeterminate sleep (transitional sleep) [27]. Newborns typically sleep for 16–18 h, with the longest uninterrupted sleep episodes lasting 2.5–4 h [25]. As individuals age, changes occur in the distribution of time spent in various sleep stages. It turns out that sleep cycles lengthen, with quiet sleep increasing, active sleep decreasing, and time spent in transitional sleep becoming less represented [28]. Typical physiological values for healthy adults are presented in Table 1.

### 2.2. Physiological Changes During Sleep

Building on the previous chapter, we begin our review of physiological changes during sleep with the most reliable indicator of sleep stages: the EEG, which is often monitored using shared electrodes with the EOG. Together with chin EMG, these signals provide a detailed understanding of sleep architecture. Next, we discuss heart rate (HR) and heart rate variability (HRV), which are among the most assessed physiological markers in wearable sleep monitoring devices. We then turn to body movement, with a particular focus on leg movements, followed by respiration—a critical physiological variable with strong associations with sleep and breathing disorders. Finally, we examine temperature and blood pressure, which also show distinct and significant variations across sleep stages.

#### 2.2.1. Electroencephalography, Electrooculography, and Electromyography

Each stage of the sleep cycle has clinical and electrophysiological characteristics. EEG, EOG, and chin EMG are essential for the accurate scoring of sleep stages, resulting in a graphical output called a hypnogram. To determine the sleep stage, it is necessary to recognise the type of EEG waves based on their frequency and amplitude, identify characteristic EEG features specific to each sleep stage, and assess eye movements and chin muscle activity. The detailed scoring rules for sleep stages are further described in the AASM Manual for the Scoring of Sleep and Associated Events [29]. This manual includes detailed guidelines for assessing individual sleep stages and evaluating respiratory events in both adults and children.

In a relaxed wakeful state with closed eyes, EEG activity presents higher frequencies compared to sleep, typically in the alpha wave range (8–13 Hz) and with low amplitude. During non-relaxed wakefulness, beta waves with a frequency of 14–30 Hz are commonly observed [30]. EOG signals are particularly effective in differentiating REM sleep from non-REM stages, because REM is characterised by rapid, jerky eye movements, whereas non-REM stages show slower or minimal eye activity. This makes EOG a useful tool for identifying these stages with high precision [31,32]. The shape of EOG signals during non-relaxed wakefulness varies depending on the activity (e.g., reading, blinking, or eye rolling). Chin muscle EMG shows sustained tonic activity with high amplitude, while respiration remains irregular [33].

Sleep stage NREM 1, also called theta sleep, is characterised by low amplitude and mixed frequency (LAMF) activity, where theta waves dominate (4–7 Hz) on the EEG [30]. In this stage, the alpha rhythm dissipates, and vertex sharp waves, lasting up to 0.5 s, become visible. Slow, conjugated eye movements (SEMs) can be observed via EOG during NREM 1. Chin EMG shows a lower amplitude of tonic activity compared to the waking state, but it is still high [33]. The transition to NREM 2 is defined by the occurrence of sleep spindles or a K-complex without arousal.

Light sleep, or NREM 2, also known as spindle or sigma sleep, is marked by theta waves (4–7 Hz) with low to moderate amplitude [30]. This stage is distinguished by the presence of sleep spindles and/or K-complexes in the EEG. Sleep spindles are brief bursts of high-frequency activity (11–16 Hz) with a spindle-shaped appearance, while K-complexes are sharp, long-lasting delta waves (~1 s), known as the largest and most noticeable brain waves. K-complexes are important for maintaining sleep and consolidating memory. Minimal or absent eye movements are characteristic of NREM 2, along with chin EMG lower than in NREM 1. This sleep stage typically lasts about 25 min during the first sleep cycle, with duration increasing in subsequent cycles, eventually comprising around 45% of total sleep. Bruxism (involuntary teeth grinding or jaw clenching) may occur in this stage [25].

Sleep stage NREM 3 is often referred to as slow-wave sleep. Slow delta waves (0.5–3.5 Hz) with high amplitude, at least 75 µV, particularly in the frontal leads, have occurred. If 20% or more of the epoch contains delta waves with an amplitude greater than 75 µV, it is classified as NREM 3. Eye movement is absent, and EMG of the chin muscle shows lower activity compared to NREM stage 2, with minimal motor manifestations. The arousal threshold is higher compared to other sleep stages [30]. Deep sleep plays a vital role in the body’s recovery, especially during periods of illness or growth. Metabolic rate reaches its lowest point [34].

REM sleep, or paradoxical sleep, is distinguished by desynchronised EEG activity, where sawtooth waves (2–4 Hz) with moderate amplitude appear in small clusters. They occur simultaneously with rapid eye movements (REMs), characteristic of phasic REM sleep. Another key feature is continuous chin muscle atonia, occasionally interrupted by brief muscle twitches. Low chin EMG activity, REMs, and LAMF without sleep spindles or K-complexes differentiate this stage from others. Respiration is irregular, and emotionally charged dreams occur [24]. REM sleep is important for brain development, learning, memory consolidation, and emotional processing [35,36]. Recent studies have also shown that single-channel EOG can be a reliable alternative to the more complex and intrusive EEG for sleep monitoring. For example, a deep-learning approach using EOG signals achieved comparable accuracy to EEG in classifying sleep stages, demonstrating its potential for home-based and clinical sleep monitoring [37,38]. This makes EOG especially valuable for diagnosing disorders like REM sleep behaviour disorder (RBD), insomnia, or circadian rhythm disruptions, as these often manifest in altered sleep-stage dynamics detectable via EOG. Additionally, EOG is more user-friendly and less invasive than EEG. Techniques like attaching electrodes to a sleep mask enable easy data collection, which is practical for long-term monitoring. In disorders like RBD, where abnormal muscle and eye movements occur during REM sleep, EOG can help detect these irregularities early, aiding in diagnosis and treatment strategies.

#### 2.2.2. Heart Rate and Heart Rate Variability

Another non-invasive method for sleep stage classification, which is associated with a wide range of sleep disorders, is HR and its variations in the form of HRV. HR and HRV are distinct yet complementary markers that provide critical insights into the autonomic nervous system (ANS) and its regulation during sleep. Each metric plays a unique role in understanding sleep architecture and related disorders. HR directly reflects cardiac activity and is particularly valuable for identifying transitions between sleep stages [39]. During NREM sleep, parasympathetic activity predominates, leading to a gradual reduction in HR. These physiological changes support cardiovascular recovery, metabolic conservation, and overall restorative processes. As sleep progresses from lighter NREM stages (e.g., NREM 1) to deeper ones, parasympathetic (PNS) tone increases, while sympathetic (SNS) tone decreases. This shift leads to reductions in heart rate, lessening the burden on cardiac output and inducing autonomic stability [40]. Upon entering REM sleep, there is a shift to SNS dominance, accompanied by abrupt increases in HR [41]. These changes are closely linked to the vivid dreaming and heightened brain activity typical of REM sleep.

The autonomic shifts observed during sleep suggest that these transitions serve essential autonomic-related functions. In conditions such as OSA, severe episodes are marked by elevated HR and sympathetic overactivation, especially during REM sleep. This overactivation contributes to fragmented sleep and significant cardiovascular stress [42]. HRV, on the other hand, offers insights into the dynamic balance between the SNS and PNS branches of the ANS [43]. The SNS, responsible for the “fight or flight” response, increases heart rate and blood pressure by releasing norepinephrine, which stimulates the SA node. This reduces HRV, as the heart rate becomes more constant and less adaptable. In contrast, the PNS promotes relaxation and conserves energy by releasing acetylcholine, slowing the heart rate and enhancing HRV through greater variability between heartbeats [44]. HRV reflects the interplay between the two systems through its low-frequency (LF) and high-frequency (HF) components. The LF component represents both SNS and PNS influence, while the HF component is mainly driven by PNS activity and is most prominent during rest or sleep. The LF/HF ratio is used to assess the balance between SNS and PNS, with a higher ratio indicating sympathetic dominance and a lower ratio reflecting parasympathetic dominance. Therefore, high HRV indicates parasympathetic dominance, while low HRV suggests sympathetic dominance.

HRV provides deeper insight into the quality of autonomic regulation during sleep. Higher HRV during sleep, reflecting PNS dominance, is associated with better sleep quality and efficient restorative processes [45]. Lower HRV suggests autonomic imbalance and is linked to sleep disturbances such as insomnia, fragmented sleep, and increased stress vulnerability. In healthy individuals, HRV follows a predictable pattern across sleep stages. It is higher during NREM sleep when PNS activity supports recovery and restorative sleep, and lower during REM sleep, which involves increased SNS activity [46,47]. Certain diseases and conditions can disrupt the balance of the ANS, leading to reduced HRV. For instance, chronic stress results in prolonged activation of the sympathetic nervous system and reduced parasympathetic activity, both of which contribute to lower HRV [48]. Similarly, metabolic disorders like Type 2 diabetes and obesity often involve chronic inflammation, which increases sympathetic activity and decreases parasympathetic modulation. In Type 2 diabetes, high blood glucose levels can cause autonomic neuropathy, damaging nerve fibres and impairing the regulation of heart rate, further decreasing HRV [49]. In neurodegenerative diseases such as Parkinson’s disease and Alzheimer’s, damage to structures like the medulla oblongata, which controls autonomic functions, leads to a decrease in parasympathetic activity, resulting in low HRV and an increased risk of cardiovascular events [50]. Similarly, in chronic kidney disease (CKD), chronic inflammation and renal stress disrupt the balance between sympathetic and parasympathetic systems, increasing sympathetic activity while reducing parasympathetic activity, ultimately leading to reduced HRV [51].

HRV offers deeper insights into autonomic regulation compared to HR, enhancing sleep stage classification. During NREM sleep, increased parasympathetic activity leads to a decrease in HR and an increase in HRV, with the highest HRV observed in N3, supporting physical recovery. Conversely, during REM sleep, sympathetic activation results in greater HR variability and lower HRV, reflecting higher physiological demands [41].

Although HR and HRV are separate metrics, their combined use in sleep research enhances our ability to understand sleep disorders and autonomic regulation. HR captures immediate cardiovascular responses, while HRV offers a more nuanced understanding of autonomic flexibility and adaptation. Together, they provide comprehensive insights into the complex relationship between sleep, ANS function, and associated pathological conditions like OSA and neurodegenerative diseases. In conclusion, the integration of HR and HRV provides a more comprehensive understanding of sleep dynamics and autonomic regulation. While HR offers immediate cardiovascular insights, HRV reveals deeper autonomic mechanisms, highlighting the body’s capacity to adapt and recover during various sleep stages. Together, these metrics offer diagnostic potential for sleep-related disorders and autonomic dysfunctions, making them valuable tools for advancing sleep medicine and personalised health monitoring.

#### 2.2.3. Body Movement

Tracking body movement during sleep is a non-invasive method for assessing sleep patterns, autonomic regulation, and overall sleep quality [52]. Movement patterns, including large body movements (LMM), vary notably across sleep stages. According to Ibrahim et al. [53], LMMs are more frequent but shorter during REM sleep compared to NREM sleep, with the lowest occurrence observed in NREM 3. The authors also noted that men exhibit higher LMM indices than women, and while the overall prevalence of LMMs remains stable with age, their likelihood of causing awakenings increases over time. In contrast, Gori et al. [54] found that body movements, including LMMs, decrease significantly in elderly individuals compared to younger subjects, suggesting a gradual reduction across the lifespan. Unlike younger adults, whose sleep-related body movements predominantly occur during REM sleep, elderly individuals showed no preferential association with specific sleep stages. These findings may reflect age-related changes in the interaction between motor cortex control and subcortical circuits. This discrepancy may arise from differences in study populations or methodologies.

LMM indices also correlate with sleep fragmentation, highlighting their potential as markers of sleep quality and restlessness. These fluctuations are valuable for detecting disruptions such as apnoea or nocturnal awakenings, which are associated with poor sleep quality, reduced efficiency, and daytime impairment [55]. Body movement also reflects autonomic regulation, with reduced movement indicating parasympathetic dominance during deep NREM sleep, and increased movement signalling sympathetic activation during REM sleep [40]. Tracking body movement during sleep provides valuable insights into sleep patterns, autonomic regulation, and sleep quality. The frequency and nature of large body movements (LMM) differ across sleep stages, with LMMs serving as indicators of sleep fragmentation and autonomic function. These movements reflect shifts between parasympathetic and sympathetic dominance, making body movement tracking a useful tool for diagnosing sleep disorders and monitoring sleep.

#### 2.2.4. Respiration

As mentioned in the introduction, many people worldwide suffer from sleep disorders that can lead to deterioration in their quality of life. These disorders can disrupt sleep patterns (depth and duration), and manifest in various symptoms such as difficulty falling asleep, snoring, awakenings during sleep, or more serious health complications. Insomnia and sleep-related breathing disorders are the most common diseases among all sleep disorders. For that reason, measuring respiratory activity is crucial for assessing sleep quality, as deviations in normal respiratory patterns can be indicative of various sleep disorders and overall sleep efficiency (SE). Normal respiratory rates (RR) at rest typically range between 12 and 20 breaths per minute (rpm) in adults [56]; however, this rate generally decreases during sleep [57]. Accurate monitoring of RR during sleep can help identify issues such as OSA, central sleep apnoea (CSA), hypoventilation and hypoxemia, and other respiratory dysfunctions, all of which significantly affect sleep quality [58]. Breathing disorders may involve interrupted breathing during sleep, caused by pauses or reduced airflow. Although frequently underestimated, sleep-related breathing disorders pose a serious threat to individual health.

One of the most frequently occurring sleep disorders is sleep apnoea, affecting 9–38% of the global population [2]. OSA alone is estimated to affect up to one billion people worldwide, predominantly in the age range of 30 to 69 years. Its prevalence continues to rise, primarily due to the global increase in obesity, a major risk factor for OSA [59]. Historically, the male-to-female ratio for OSA prevalence was around 4:1, but it is now believed that many women with OSA have been underdiagnosed [60]. The epidemiology of CSA has been studied in less detail. CSA is estimated to affect 5% to 10% of individuals with sleep-related breathing disorders [61]. Untreated OSA, which is associated with rhythm disturbances, serves as an independent predictor of both systemic and pulmonary hypertension and more than doubles the risk of heart failure. Such patients have elevated incidences of ischemic heart disease and stroke, and OSA contributes to metabolic disorders by affecting endocrine regulation [5]. Common symptoms include intermittent snoring, breath-holding, frequent awakenings with gasping, nocturia, excessive daytime sleepiness, unrefreshing sleep, morning fatigue, and issues with concentration. Furthermore, sleep apnoea can impact driving safety, as sleep interruptions may lead to microsleeps or impaired reaction times, increasing the risk of accidents on the road. Therefore, early diagnosis is important for patient safety and well-being [30].

In the context of sleep-related breathing disorders, the following terms are often encountered: Apnoea is defined as a respiratory event lasting more than 10 s with a reduction in airflow amplitude of ≥90%. A hypopnoeic respiratory event is defined as an event lasting more than 10 s with a reduction in airflow amplitude of ≥30%, associated with a desaturation of at least 3% and/or an arousal (a brief period of wakefulness lasting 3–15 s) [62]. Desaturation refers to a drop in oxygen saturation by at least 3%, or 4% in some cases. It is recorded by pulse oximetry and is commonly associated with apnoeic and hypopnoeic events. Desaturation can also be present in patients with hypoventilation [33]. The number of desaturations per hour of sleep is expressed by the oxygen desaturation index (ODI). The severity of obstructive sleep apnoea/hypopnoea syndrome in adults is classified using the apnoea–hypopnoea index (AHI), which represents the number of respiratory episodes per hour of sleep (Table 2) [5].

Individual respiratory events are categorised based on the presence or absence of respiratory effort, which is usually detected through respiratory belts placed around the thorax and abdomen. Obstructive sleep apnoea/hypopnoea is characterised by an obstruction in the upper airways and involves preserved respiratory effort. In contrast, central sleep apnoea/hypopnoea is caused by malfunction in the central nervous system, defined as a respiratory event with no respiratory effort. Mixed sleep apnoea/hypopnoea is identified as a respiratory event with an initial absence of respiratory effort, followed by the recovery of effort during the event’s duration [10,62].

In summary, monitoring respiratory activity during sleep is essential for detecting abnormalities and assessing sleep efficiency. The classification of respiratory events, including apnoea, hypopnoea, and desaturation, plays a critical role in diagnosing and managing conditions like OSA and CSA. Poor sleep caused by respiratory diseases can have negative effects on daily life, including excessive daytime sleepiness and impaired cognitive function. These diseases can also have a negative impact on cardiovascular health. Therefore, early detection of sleep-related respiratory diseases is crucial for maintaining a patient’s long-term health and quality of life.

#### 2.2.5. Body Temperature

During the day, skin body temperature tends to fluctuate slightly between 1 and 2 °C. The temperature reaches its lowest values in the morning and its highest values in the early evening. This is the so-called circadian rhythm, which is influenced by melatonin [64]. During sleep, the core body temperature (CBT) may be 0.4–0.6 °C lower than during the day. Body temperature starts to fall just before the onset of sleep, which prepares the body for night sleep [65]. The extent of the decrease in CBT just before sleep corresponds to the onset and quality of sleep. A decrease in CBT is also observed during the duration of sleep, with the lowest temperature being reached in NREM 3 sleep [66]. In contrast, body temperature increases during REM sleep [67]. By manipulation of the body temperature, it is even possible to increase sleep quality [68,69,70], or initiate NREM sleep [71].

It can be said that body temperature is deeply connected to the sleep cycle. These temperature fluctuations not only indicate the quality of sleep, but by gently manipulating the temperature, we can also improve it. A key limitation in measuring CBT is its susceptibility to external factors such as ambient temperature and bedding. To improve accuracy, sensors should be placed on the chest rather than the wrist, and room temperature should be monitored to adjust CBT estimations accordingly.

#### 2.2.6. Blood Pressure

During healthy sleep, there is a physiological decrease in systemic BP compared to wakefulness. This nocturnal decrease is referred to as “dipping” and is attributable in part to a reduction in SNS activity. NREM is linked with stabilisation of BP in healthy individuals, and as sleep shifts from NREM 1 to NREM 3, there is an approximately 5–14% reduction in arterial BP and peripheral vascular resistance.

Blood pressure in REM sleep is thus about 5% higher compared to NREM sleep. A decrease of 10% to 20% in average nocturnal BP (both systolic and diastolic) compared to average daytime BP is considered normal. Conversely, the absence of nocturnal dipping is referred to as a <10% reduction in nocturnal BP [67]. Reduced nocturnal BP dip is a strong, independent predictor of cardiovascular risk [72]. There is a sharp rise in BP during waking. Pulmonary arterial pressure rises slightly during sleep. Its mean value is 18/8 mmHg during wakefulness and 23/12 mmHg during sleep [67].

Healthy sleep is characterised by a decrease in blood pressure due to reduced sympathetic nervous system activity, but REM sleep shows a slight increase of it. The absence of a nocturnal decrease is an indicator of increased cardiovascular risk. Blood pressure monitoring is less commonly used in sleep studies, primarily because standard certified methods rely on cuff-based measurements, which can be disruptive during sleep. A promising alternative is cuffless monitoring using PPG and ECG signals to estimate pulse transit time. While its absolute accuracy is still limited, it is sufficient for tracking blood pressure variations.

To provide clarity, we have compiled a concise overview of the key physiological changes during sleep in Table 3.

### 2.3. Altered Sleep Physiology in Neurological Diseases

The relationship between sleep and neurodegenerative diseases is not yet well understood, but it is certain that they are closely related. Sleep disorders are common in these diseases, and investigations of how this fact can be used to slow cognitive decline and for early diagnosis are ongoing. Diseases such as AD and PD begin before they manifest as either movement disorders (PD) or forgetfulness (AD), and the search for symptoms that occur in the early stages is very important to improve the quality of life of patients [74,75,76].

In its conventional diagnosis, PD has traditionally been recognised as a movement disorder based on motor features such as bradykinesia, rigidity, and tremor. Nonmotor symptoms have become increasingly important in recent years, as they frequently result in hospitalisation and increase the cost of patient care and may be an earlier indicator of the disease than movement problems. Sleep disorders certainly have clinical significance. Insomnia, daytime sleepiness, restless leg syndrome, and RBD affect more than 90% of PD patients and worsen over the course of the disease; OSA is also frequent in PD [75,77]. For example, a loss of dopaminergic neurons in the substantia nigra was found in the brains of people with PD, and RBD could serve as an early sign of changes in dopaminergic neurotransmission [78]. In fact, many patients with RBD who have been monitored have developed a neurodegenerative disorder [79]. PSG analyses demonstrate a beneficial role of NREM 3 [75,80] and suggest that REM sleep may help maintain neuronal homeostasis because its disruption leads to neurodegeneration [81]. Determining the exact changes in PSG in PD patients and controls is not straightforward, as the study groups are highly heterogeneous in different studies, and age or sex can have varying effects. Sleep duration in middle-aged adults is a predictor of age at diagnosis of PD, and short duration (<7 h) is associated with a younger age at diagnosis of PD [82]. Chronic short sleep duration (<7 h per day) reduces the brain’s ability to clear the accumulation of toxins and proteins, which may contribute to PD as well as AD. The study by Tanaka et al. provides insight into the number of turns during sleep, contending that reduced sleep activity could be an early indicator of PD (specifically, fewer than six turns per night) [83]. Reduced turning frequency may precede muscle weakness. Specifically, PSG studies have been conducted by Yong et al. [84], who used PSG to investigate sleep disorders in PD and conducted one of the largest case-control studies, involving overnight polysomnographic evaluations of 56 PD patients and 68 healthy controls. The analysis showed that patients had shorter sleep time, lower sleep efficiency, and increased REM latency.

Tracking body movements during sleep is crucial for diagnosing and monitoring sleep disorders such as periodic limb movement syndrome (PLMS) and restless leg syndrome (RLS), and for evaluating patients with neurodegenerative conditions like PD and AD. In patients with PD and AD, symptoms such as restless sleep, movements during sleep, and repetitive leg movements often indicate sleep-related disorders. Monitoring these movements can not only aid in early diagnosis but also serve as a tool for assessing the effectiveness of therapeutic interventions aimed at improving sleep quality [17,85].

Monitoring EMG activity during sleep can detect subtle muscle activity that often precedes overt physical movements, facilitating early diagnosis of RBD [86]. This is particularly important because RBD is strongly associated with neurodegenerative disorders, especially PD. Studies indicate that a significant proportion of individuals diagnosed with idiopathic RBD go on to develop PD or other synucleinopathies, such as dementia with Lewy bodies or multiple system atrophy, over time [87,88,89]. Identifying RBD early through EMG monitoring allows for closer neurological follow-up and may provide a critical window for early interventions, potentially slowing the progression or mitigating the impact of associated neurodegenerative conditions. Especially with automatic identification from data from polysomnography and EMG, this identification can be faster and free from manual scoring bias [90].

In AD patients, the disease causes the accumulation of amyloid-ß protein, which aggregates into plaques, and later tau protein, which leads to atrophy of key brain regions. It has been found that insufficient deep sleep is associated with early symptoms, and in older people who sleep poorly, increased amounts of tau protein are found. PSG studies of sleep changes in AD have suggested, but not fully demonstrated, a relationship between sleep disturbances and AD. Overall, studies show that better sleep is associated with lower risk or slower progression of AD [91]. Ju et al. [22] found that Aβ deposition in preclinical AD before the onset of cognitive impairment was associated with poorer sleep quality, and frequent napping was also associated with amyloid deposition, but no changes in sleep quantity were found. Patients experience significant reductions in total sleep time (TST), sleep efficiency, and percentage of slow-wave sleep (SWS) and REM sleep, and conversely, increases in sleep latency, wake time after sleep onset, and number of awakenings. Reduced SWS and REM significantly correlate with the severity of cognitive impairment in patients [92]. Disrupted SWS activity significantly increases amyloid-ß protein levels. REM sleep helps maintain neuronal homeostasis in the brain, and its disruption negatively affects neurogenesis, and its loss likely leads to neurodegeneration and increased tau protein levels. [93,94]. Changes in EEG components and sleep spindles have also been observed. It has been found that insufficient deep sleep is associated with early symptoms, and that increased amounts of tau protein are found in older people who sleep poorly [95]. Pulver et al. [96] show that EEG recording is important for early diagnosis of AD because neural circuits associated with memory generate oscillatory events including theta bursts (TB), sleep spindles (SP), and slow waves (SW), and in AD there are changes in these events, with reduced TB spectral power in SW–TB connections and lower accuracy in SW–SP connections compared to amyloid-negative individuals. Disturbed nighttime sleep, characterised by restlessness and subsequent daytime fatigue, may signal a future diagnosis of AD. Roh et al. [97,98] reported that after the formation of amyloid-ß plaques in a mouse model, the sleep–wake cycle is disrupted, and after their removal, the cycle returns to normal, demonstrating a link between neurodegeneration and sleep. The plaques are present in the brain long before symptoms appear. Gaeta et al. [99] conducted a PSG study in patients with mild to moderate AD, also testing cerebrospinal fluid (CSF) and blood samples for biomarkers. They used a multimodal machine learning (ML) approach. The results showed that multimodal ML can help predict the outcome of CSF biomarkers in early AD, the impact of hypoxemia on higher CSF amyloid levels, and hypopnoea and apnoea events associated with levels of pathological AD markers and cognitive decline. Tao et al. [100] used baseline PSG data from mild-to-moderate AD patients and older healthy controls, with AD patients showing a lower percentage of time spent in slow-wave sleep (and a correspondingly higher percentage of time spent in lighter NREM 1 sleep), lower spindles per minute of NREM 2 sleep, and lower absolute EEG power during NREM sleep, particularly in the low-frequency bands.

Neurological disorders, including synucleinopathies, disrupt the balance of the ANS, leading to reduced HRV and atypical sleep patterns. These reductions are often associated with degeneration in brain regions responsible for autonomic regulation, such as the brainstem and cortical areas. HRV analysis during sleep can provide valuable insights into biomarkers for disease progression, therapeutic targets, and mechanisms underlying autonomic dysfunction. It may also serve as an early indicator of complications in neurodegenerative diseases.

In PD, impaired HRV has been associated with disease severity, the duration of motor symptoms, and the dosage of dopaminergic medications [101,102]. Devos et al. [103] also observed progressive nocturnal cardiac dysregulation as PD advances. Specifically, the more advanced the PD, the lower the high-frequency (HF) HRV components and the higher the low-frequency/high-frequency (LF/HF) ratio. This pattern indicates diminished vagal output and increased sympathovagal balance during sleep. HRV patterns also appear to distinguish PD patients with RBD from those without it, supporting HRV’s potential as a digital biomarker [104]. In AD, studies [105,106] indicate reduced parasympathetic activity, reflected in low HF-HRV during SWS, particularly in older adults at risk for dementia. This reduction is most pronounced in individuals with amnestic mild cognitive impairment (MCI), a precursor to AD. These findings suggest that parasympathetic activity during SWS might serve as an early biomarker of neurodegeneration, providing peripheral evidence of underlying pathological processes [106]. Both AD and PD are characterised by reduced HRV during sleep, but their underlying mechanisms differ. These distinctions highlight the role of HRV as a non-invasive tool for understanding the pathophysiology of neurodegenerative diseases, particularly during REM and non-REM sleep stages.

There are multiple sleep disorders associated with neurodegeneration, and accurate diagnosis can be challenging, but through the use of home PSG and appropriate algorithms, it may be possible to capture these diverse symptoms and detect specific disorders [76]. However, there is also heterogeneity among studies, and thus further research is needed to identify specific early markers of neurodegenerative diseases to ensure high patient coverage. Overall, several studies of PSG have been conducted on AD and PD patients which indicate the suitability of PSG for monitoring patients with neurodegeneration and early manifestations of these diseases. The specific factors are in the research phase, but they hold great promise for early detection of diseases and early treatment.

## 3. Current State of Technological Evolution

**Figure 2:**
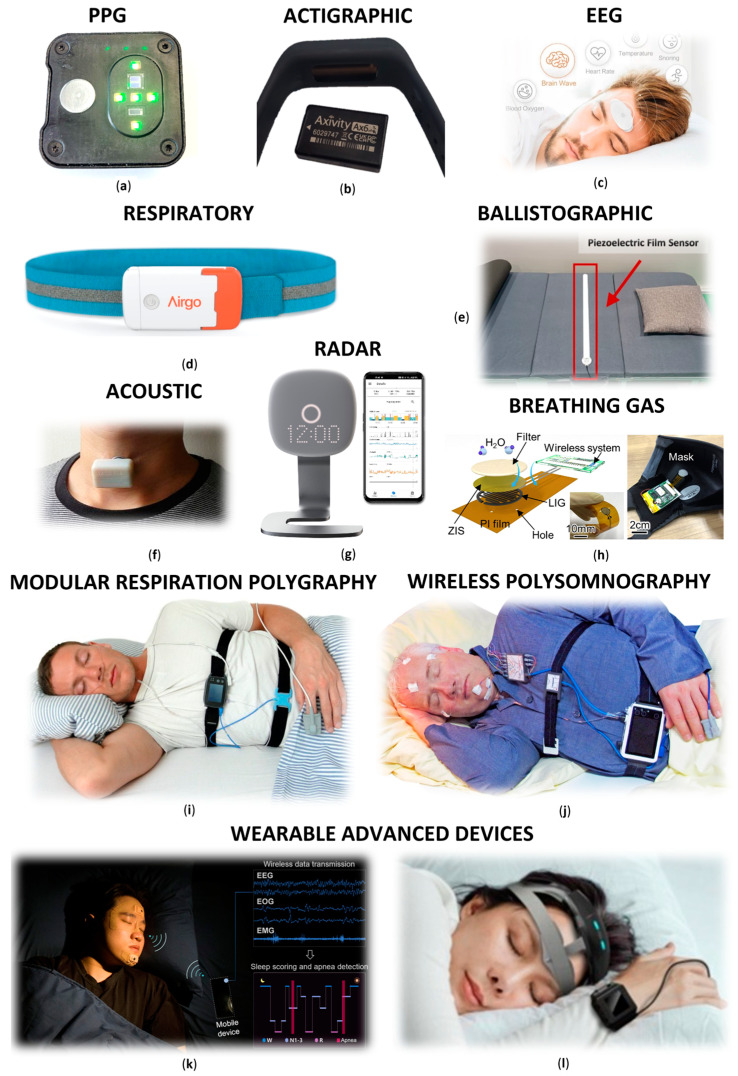
Interesting examples of the current state of basic sleep monitoring devices according to technologies: (**a**) ePPG—our own multi-sensor design with PPG, temperature, and movement sensors for sleep analysis used for PD studies [10]; (**b**) SleepActa actigraphic monitor with Dormi algorithm [107]; (**c**) UMindSleep EEG-based sleep tracker, reprinted from ref. [108]; (**d**) Airgo chest belt for monitoring sleep respiration disorders, reprinted from ref. [109]; (**e**) piezoelectric BCG sensor placed under bed mattress, reprinted from ref. [110]; (**f**) wearable apnoea acoustic detection device, reprinted from ref. [111]; (**g**) Somnofy radar system for contactless analysis of sleep stage and RR, reprinted from ref. [112]; (**h**) flexible humidity sensor in facemask for sleep apnoea monitoring, reprinted from ref. [113]; (**i**) modular respiratory polygraphy device SOMNOtouch^TM^ RESP (©2025 SOMNOmedics GmbH), reprinted from ref. [114]; (**j**) wireless PSG device SOMNO HD (©2025 SOMNOmedics GmbH), reprinted from ref. [115]; (**k**) wireless wearable sleep monitoring patches, reprinted from ref. [116]; (**l**) advanced wearable headband and wrist device WPSG-I, reprinted from ref. [117].

The field of sleep monitoring devices has evolved significantly due to technological advances and the growing consumer demand for accessible health insights (Figure 2). This chapter systematically reviews these technologies, starting with the most widespread and progressing towards more specialised approaches, reflecting their current role in the emerging field of sleep monitoring. Each section highlights the unique capabilities, limitations, and advances of these devices, highlighting their impact on consumer health and clinical diagnostics.

Overall, the chapter is structured into three subchapters. The first focuses on basic devices that rely primarily on a limited range of physiological parameters, offering a simple approach to sleep monitoring (Table 4). The second subchapter explores more advanced systems, which utilise multiple physiological parameters and can be considered as variants or extensions of traditional PSG technology (Table 5). Finally, the third subchapter delves into research applications specifically targeting neurological disorders, often employing devices described in the previous sections.

### 3.1. Basic Sleep Monitoring Devices

#### 3.1.1. PPG-Based Devices

Photoplethysmography (PPG) is a non-invasive method based on optical measurement of volume changes in the blood circulation. The main advantages of PPG are simplicity and low cost. PPG signals can be sensed and recorded from various body points such as the wrist, finger, ear, nose, forehead, arm, neck, etc. [83,118,119,120,121,122,123]. PPG uses a light source (LED) and a photodetector (PD) to record volume changes in blood circulation related to variations in light absorption, thus providing information about HR and pulse oximetry values and enabling the monitoring of various cardiovascular diseases. The incorporation of this method is very popular on wrists or fingers [124], mainly in smart watches like Apple watch 10 (Apple Inc., Cupertino, CA, USA), Xiaomi Mi Band 9 (Xiaomi, Beijing, China), FitBit Sense 2 (Google Inc., Mountain View, CA, USA) [124,125,126,127,128,129], smart rings (Oura Ring (Oura Health, Oulu, Finland), O_2_ Ring (Wellue, Shenzhen, China), RingConn Gen 2 (RingConn LLC, Wilmington, DE, USA) [130,131,132], and various other multi-sensor devices for measuring human physiology that are also suitable for continuous measurement [133]. The use of PPG sensors for sleep monitoring is interesting for its ability to capture the modulation of the autonomic nervous system during sleep. The combination of PPG with accelerometery helps to construct hypnograms in sleep and detect sleep-disordered breathing (SDB) [134]. The combination with other sensors also seems to obtain results, for example in conjunction with brain activity. Using PPG and a suitable algorithm, it is possible to detect the onset of sleepiness approximately 9 min before sleep onset by analysing the change in the LF/HF parameter [135] or sleep stage in conjunction with body movements. The use of PPG for sleep monitoring is suitable for home long-term monitoring of insomnia, circadian rhythm sleep disorders, and treatment of SDB and OSA [134,136,137].

One of the most precise PPG-based sleep trackers currently available is the Oura Ring Generation 3 and 4. Using ML algorithms and a dataset of over 1200 PSG-validated recordings, the Oura Ring achieves 79% agreement with PSG [138], approaching the reliability of human experts, which has been 83% [139] and 88% [140], in sleep stage scoring. The Oura Ring uses HR and movement data to classify sleep into light, deep, REM, and waking stages. Its IR PPG sensors allow for deeper tissue monitoring, providing more accurate physiological measurements, such as HRV, compared to devices which rely on green light for more superficial signal capture. While IR light is more prone to movement artifacts, the Oura Ring compensates for this by integrating movement data. Green light, commonly used in other devices, offers a better signal-to-noise ratio [141], but lacks the depth of IR light, making it less effective for certain physiological assessments. The Oura Ring 4 features an 18-path multi-wavelength PPG system that improves accuracy. In contrast, the Oura Ring 3 uses a single/dual-wavelength PPG system, which is effective but less precise, particularly given challenging conditions like movement or skin tone variations. Additionally, the Oura Ring features a negative temperature coefficient (NTC) sensor to directly monitor nighttime skin temperature. Although these data are not used for sleep stage classification, they provide valuable insights into recovery and illness. A study by Robbins et al. [142] supports the Oura Ring’s accuracy in sleep tracking compared to other methods. A study by Svensson et al. [143] validated the accuracy of Oura’s Sleep Staging Algorithm 2.0, showing that its measurements closely matched PSG, with sleep staging accuracy ranging from 75.5% for light sleep to 90.6% for REM sleep. Attention should also be given to the related commentary by Lolli et al. [144], which highlighted minor inconsistencies. Tisyakorn et al. [145] screened for moderate to severe OSA with an O_2_ ring [130]. The study included 190 participants with an AHI of 50.4 and compared this with standard PSG. The optimal cutoff for 11% ODI was 1.25 events/hour lasting 20 s. They achieved a sensitivity of 87.30% and a specificity of 78.70%. The area under the receiver operating characteristic curve for identifying OSA was 0.91. The SVM (support vector machine) model demonstrated a high sensitivity of 97% in screening moderate to severe OSA but had a low specificity of 50%.

Another PPG-based device is the WHOOP 4.0 (Whoop, Boston, MA, USA). WHOOP’s PPG system includes three green LEDs which enhance the accuracy of heart rate measurements, one red LED for SpO_2_ monitoring, and one IR LED for tracking HRV. WHOOP also uses PPG data to estimate RR and calculate HRV. In addition to PPG, WHOOP 4.0 integrates data from multiple sensors, including a 3D accelerometer and gyroscope that detect movement and body orientation. These sensors, combined with PPG data (HR, HRV, resting HR, and RR), offer a more detailed analysis of sleep patterns and stages. Like the Oura Ring, WHOOP features a temperature sensor, though it does not use NTC technology. Instead of sleep stage classification, WHOOP monitors ambient and skin temperature primarily for assessing stress and recovery. Although WHOOP 4.0 has not yet been extensively validated in peer-reviewed studies, a study published in [146] supports the accuracy of WHOOP 3.0 in classifying sleep stages. WHOOP claims that the 4.0 model offers a 10% improvement in accuracy compared to the 3.0 version, largely due to enhancements in sensor technology, including the addition of SpO_2_ and skin temperature sensors [147]. Among PPG-based sleep trackers, such as the Samsung Galaxy, Apple Watch, Garmin, Xiaomi Mi Band 5, and Google’s Pixel Watch, Fitbit stands out as one of the most well-validated options, particularly the Sense 2 and Charge 5 [148]. These models use advanced algorithms based on HR and movement data (via accelerometer) to classify sleep into a simpler three-stage system: light, deep, and REM sleep. While Fitbit tracks HRV**,** it does not use HRV directly for sleep stage classification. Instead, HRV contributes to evaluating sleep quality**,** recovery**,** and overall health. Fitbit uniquely provides explicit SpO_2_ metrics, which help identify breathing irregularities potentially linked to sleep apnoea, though this information is presented as trends rather than direct alerts. The Sense 2 also includes an electrodermal activity (EDA) sensor**,** which helps address stress-related sleep disruptions, indirectly improving sleep quality. Additionally, both models monitor nightly skin temperature variations. Another device is the UpNEA [124], which is in the form of a smart glove. It contains a three-axis accelerometer on the wrist connected to a PPG sensor on the finger. The device is mainly suitable for determining sleep stages, apnoea, and hypopnoea, but of course it can also identify HR, SpO_2_, RR, and atrial fibrillation. The apnoea and hypopnoea detection algorithm showed an accuracy of 75.1% when displaying the PPG window in one-minute segments. From the accelerometer, the device can distinguish CSA from OSA with an accuracy of 92.6%, central hypopnoea from CSA with an accuracy of 83.7%, and OSA from obstructive hypopnoea with an accuracy of 82.7%.

The devices described so far have been used on the wrist or finger. Young children and babies are often monitored through their feet. Regarding the application of PPG in less traditional locations, it is worth mentioning the study of Venema et al. [149], which explored PPG sensors worn in the ear canal. The authors highlighted the reliability of home measurements without the need to conduct all measurements in laboratory conditions, comparing sensor results with standard PSG monitoring. They diagnosed sleep apnoea and evaluated the dynamics of HR and SpO_2_ and discussed methods for deriving RR from PPG signals. Another study [150] utilised a device placed at the root of the nose for home all-night screening of sleep-disordered breathing, called Morfea. This device is designed to detect sleep apnoea and assess various sleep parameters. Morfea contains a PPG sensor with two LEDs, a microcontroller, a 3D accelerometer, a Bluetooth unit, and a battery with a guarantee of 9 h of data acquisition. The recorded PPG signal is filtered using a band-pass filter with a passband frequency range of 0.3 Hz to 3.5 Hz to preserve the cardiac and respiratory components and remove high-frequency noise. Morfea is effective in detecting sleep apnoea and can also identify five different body positions during sleep, estimate SpO_2_, which is a direct indicator of sleep apnoea, measure HR, and determine the severity of sleep-disordered breathing. The device’s limitations include its inability to distinguish between apnoea and hypopnoea and its inability to classify sleep stages. The study results show an 89% sensitivity and 93% accuracy in detecting sleep apnoea. Also of note is the review by Perez-Pozuelo et al. [151] dedicated to sleep detection outside the clinic using wearable HR measurement devices.

Measurements using PPG sensors provide very valuable information about the overall physiological state of the patient and, in addition to HR, RR, and SpO_2_, pulse oximetry can allow the measurement of other vital parameters such as thermoregulation or blood pressure fluctuations, thus reducing the number of sensors on multi-sensor devices.

#### 3.1.2. Actigraphic Devices

Accelerometers, as part of actigraphy devices, are usually made in the shape of wristbands or anklets and are focused on detecting cycles of sleep or wakefulness. They do not provide detailed information about sleep stages but are acceptable and effective tools for assessing disorders related to sleep patterns [152] such as insomnia and circadian rhythm disorders [153]. Sleep interpretation from actigraphy assumes that little or no movement is registered during sleep, while wakefulness corresponds to higher movement activity. It quantifies movement exceeding a predetermined threshold [154]. When an actigraphy device is placed on a foot [155], it is possible to detect RLS or PLMS in sleep. The device can determine the duration, amplitude, and periodicity of movements, as well as the severity of PLMS.

ActTrust 1 and ActTrust 2 (Neurocare Group AG, Munich, Germany) [156] are typical wrist-worn actigraphy systems. These devices enable the estimation of various objective sleep parameters, including time in bed (TIB), wakefulness after sleep onset (WASO), sleep onset latency (SOL), TST, and SE. They contain an accelerometer, temperature sensors for measuring skin and ambient temperature, as well as an RGB light sensor and an IR sensor for monitoring environmental light exposure. The battery in these devices is rechargeable and allows for monitoring over long periods, up to three months, on a single charge. For advanced monitoring, the ActLumus (Condor Instruments, Sao Paulo, Brasil) device [157] has been developed, which additionally includes photopic and melanopic light sensors for light exposure measurement. It offers 10 light channels and features off-wrist capacitive sensor monitoring. Another actigraphy system, the ActiGraph wGT3X-BT (ActiGraph LLC, Pensacola, FL, USA) [158], is a proven wearable device utilised by researchers worldwide for continuous, real-world monitoring of sleep and activity. This device excels in tracking various metrics, including physical activity (total movement, step count, energy expenditure, etc.) and estimation of basic sleep parameters mentioned above. It can communicate via Bluetooth LE, enabling the monitoring of parameters such as HR. Hayano et al. [159] performed quantitative detection of sleep apnoea using an inertial measurement unit (IMU) embedded in wristwatch devices. In their study, 122 adults underwent parallel PSG examinations. They operated with both accelerometric and gyroscopic signals and developed an algorithm to extract signals in the respiratory frequency band (0.13–0.70 Hz) and detect respiratory events as transient (10–90 s) decreases in amplitude. The respiratory event frequency correlated with AHI of the PSG with *r*  =  0.84, and the accuracy of detection was 85% for moderate apnoea and 89% for severe apnoea. One of the promising algorithms used for sleep analysis in wearable sleep trackers is Dormi (SleepActa, Pisa, Italy) [107,160]. Dormi uses a neural network to process raw data from lightweight, non-intrusive wearable activity trackers typically designed for tracking physical activity. Actigraphs using the Dormi artificial intelligence algorithm assess sleep quality and duration over a 24 h circadian cycle. Actigraphic reports from SleepActa calculate and provide sleep parameters essential for analysing sleep, such as TST, SE, WASO, and sleep regularity index. Dormi is a CE-certified Class I medical device.

Modern actigraphy systems use a combination of PPG sensors, temperature sensors, gyroscopes, and barometers to provide comprehensive insight into a person’s sleep [161]. By integrating multiple sensor types, these advanced actigraphy systems can monitor various physiological parameters, such as HR, body temperature, and movement, allowing for a more detailed analysis of sleep quality and patterns. A good example is the Somno-Art^®^ [162,163], which utilises a 3D accelerometer to monitor movements and PPG to measure HR for determining all sleep stages. This medical device, certified with a CE mark, consists of an armband that collects data and standalone software equipped with AI algorithms for automatic sleep analysis, producing a corresponding hypnogram. It uses Bluetooth technology for wireless communication, enabling seamless data transfer. Scientifically validated studies have shown that its outcomes are comparable to PSG. The device achieved a sleep–wake detection accuracy of 87.8%, with a sensitivity of 93.3% and a specificity of 69.5%. The overall accuracy for detecting all sleep stages, including NREM 1, NREM 2, NREM 3, REM, and wakefulness, was 68.5%, based on a sample of 246 patients in a comparison with traditional PSG [163]. Finally, we must highlight an article dedicated to algorithms in actigraphy [164] and one on the combination of actigraphy with PPG [165].

#### 3.1.3. EEG-Based Devices

While most wearables are designed for practical use on the wrist or finger, several specialised devices focused specifically on sleep monitoring, utilizing EEG technology, are worn by different means in the head area. Due to its direct sensing of the brain activity, EEG is considered the most accurate in sleep tracking (capable of identifying all stages of sleep) and most reliable in disorder diagnosis. A disadvantage of conventional medical devices consists of the large number of monitoring electrodes and their time-consuming setup, which can disrupt natural comfort and affect the results. Modern telemedicine devices are focused on making implementation, self-application, and usage more accessible [152].

Among the head accessories, headbands like the Muse S (InteraXon Inc., Toronto, ON, Canada) [166] are very popular. In addition to EEG data, this type of headband is capable of monitoring HR, movement, position, and breathing patterns, offering a comprehensive picture of sleep tracking. In comparison, the Dreem 3 (Beacon Biosignals, Boston, MA, USA) [167] integrates EEG data with these metrics to provide detailed insight into sleep quality. By using machine learning algorithms to analyse brainwave data, it helps to track sleep architecture and diagnose any shown disturbances [168]. Similarly, the lightweight forehead monitor UMindSleep (EEG Smart, Shenzhen, China) [169] is able to evaluate sleep records and diagnose disorders, such as OSA. It can also record snoring, forehead temperature, body movement and position, HR, and SpO_2_. The captured EEG signal and SpO_2_ data are divided into 30 s epochs. After EEG artifact removal, every epoch is classified into one of the four sleep stages (via the key frequency band) and compared against PSG standards. Additionally, the algorithm identifies oxygen desaturation events (where SpO_2_ drops ≥ 3 % for ≥10 s) and calculates oxygen desaturation index (ODI). Another design introduced a convenient ear monitor [170], which makes its use barely noticeable. The structure is composed of memory foam and flexible electrodes. Its highly elastic foam can detect signals caused by physical deformation of ear canal walls. The algorithm works similarly to the above, segmenting the signal into 30 s epochs, and then analysing and classifying into the five sleep stages. For training, the authors used a publicly available EEG dataset, and for verification, they manually captured and scored PSG. Finally, there was a successfully tested set [171] with forehead EEG (and EOG) electrodes and chin EMG electrodes. All these setups have shown high consistency with standard polysomnography in terms of total sleep time, sleep efficiency, and latencies, although there are some differences in sleep stage measurements [172].

#### 3.1.4. Respiratory-Based Devices

Chest belts, whether in their traditional form or as smart patches, are highly effective for respiratory monitoring during sleep due to their ability to provide continuous, accurate, and non-invasive measurements of thoracic and abdominal movements. They are particularly useful for identifying respiratory patterns and disruptions, which are critical for diagnosing sleep-related breathing disorders such as OSA.

The Airgo belt (MyAir Inc., Boston, MA, USA) [109], for instance, uses a resistance-based sensor positioned at the lower ribcage to detect changes in chest circumference. The belt itself is made from stretchable materials with silver-coated yarn. The Airgo belt incorporates Bluetooth and can process both live and recorded data. The device also includes an IMU for activity and position detection. The patented algorithm identifies the minimum and maximum points of each breath cycle. These two points are connected and transformed into a vector, defined by its length (representing tidal volume), its baseline (reflecting punctual functional residual capacity), and its shape, which indicates the degree of upper airway patency. In one study [173], the Airgo belt was used for sleep monitoring of 120 patients and compared with respiratory sleep monitor Nox T3 (Nox Medical, Alpharetta, GA, USA) [174]. Results showed that the Airgo belt was able to classify OSA patients at different stages with 95.8% accuracy. The study by Wu et al. [175] proposed a chest belt based on respiratory inductive plethysmography (RIP) technology, specifically aimed at continuous sleep monitoring. In this study, two RIP belts were integrated into a suit to enhance comfort. Additionally, new signal processing algorithms were developed for RR extraction. Results from experiments on 10 healthy subjects showed a relative error of 15% when comparing the data with the commercial device BIOPAC MP150 (BIOPAC Systems Inc., Goleta, CA, USA). The chest belt portability and digital design make it suitable for both clinical and home environments, where it can support the detection of sleep-related respiratory disorders. Hernandez et al. [176] developed a wireless, real-time, battery-operated system for monitoring respiratory effort and body position, using an IMU sensor placed on an elastic belt. This system employs data fusion techniques to monitor respiratory effort in both supine and lateral recumbent positions. The extended Kalman filter (EKF) recursive algorithm was used, initially estimating the best value in the least-squares sense using measurements from one sensor (e.g., a gyroscope) and then refining this estimate by incorporating data from another sensor (e.g., an accelerometer or magnetometer). The device was compared with a standard respiratory belt and was validated through the Pearson correlation coefficient (PCC), with an average PCC of 0.963. Limitations include a restricted sample size of only one healthy subject due to ethics approval constraints. For better accuracy assessment, more testing subjects are needed. Another study conducted by Kristiansen et al. [177] investigated a low-cost strain gauge respiration belt called Flow, used in combination with a convolutional neural network (CNN) for sleep apnoea severity estimation. The study involved 29 subjects undergoing unattended sleep monitoring at home, using the Flow respiration belt and the Nox T3 device simultaneously. The results demonstrated an accuracy of 0.7609, sensitivity of 0.7833, and specificity of 0.7217.

New alternatives to chest belts could be smart sensor patches. In a study by Selvaraj [178], a wireless patch sensor, VitalPatch (VitalConnect, San Jose, CA, USA), was used for monitoring the sleep architecture of 42 volunteers, comparing results with standard PSG. VitalPatch is an FDA-approved, disposable device capable of measuring single-lead ECG, HR, HRV, skin temperature, body position, fall detection, and respiratory rate. The results showed an accuracy of 80.5 ± 8.3% and a Cohen’s kappa of 0.50 ± 0.18 in three-class sleep stage classification. Zavanelli et al. [179] created a wireless soft patch capable of measuring seismocardiography (SCG), ECG, PPG, and derived parameters such as SpO_2_;, HR, respiratory effort, and RR. The patch consists of a flexible circuit on an elastomeric membrane and features integrated nanomembrane electrodes. The study used machine learning for sleep staging and apnoea detection. For sleep staging, a single-layer feedforward neural network (FFNN) with 120 neurons was trained on 262 features from physiological signals and actigraphy, achieving 82.4% accuracy in classifying waking, NREM, and REM stages. For apnoea detection, a residual convolutional neural network (RCNN) was used, reaching 100% sensitivity and 95% precision using fourfold cross-validation on data from nine subjects.

A very useful alternative method for measuring respiration during sleep is bioimpedance measurement. This method is gaining popularity mainly due to its integration into biopotential transducers, such as the circuit series ADS129xR [180], AFE4960 [181], and AFE4500 [182] (Texas Instruments, Dallas, TX, USA), as well as ADAS1000 [183] and MAX30001 [184] (Analog Devices, Wilmington, MA, USA). Among the applications in the field of sleep monitoring, research by Van Steenkiste et al. [185] is worth mentioning. They introduced a novel wearable device called ROBIN, designed to measure impedance changes during breathing, along with ECG and acceleration measurements. For automated sleep apnoea event detection, a two-phase long short-term memory (LSTM) deep learning algorithm was implemented. The study involved 25 patients, with their vital signs simultaneously recorded using a bioimpedance sensor and standard PSG. The results demonstrated that the device achieved an accuracy of 72.8%, sensitivity of 58.4%, and specificity of 76.2%.

Chest belts, smart patches, and bioimpedance-based devices each offer unique advantages and limitations for respiratory monitoring during sleep. Chest belts provide continuous and accurate tracking of thoracic movements, making them highly effective for detecting sleep-related breathing disorders. However, they may cause discomfort over prolonged use, especially if tightly fitted, and their accuracy can be affected by body position and motion artifacts. Smart sensor patches, on the other hand, improve wearing comfort by eliminating restrictive straps while offering multi-parameter monitoring, including ECG, SCG, and HR. Their major drawback is limited adherence over long periods and potential signal degradation due to skin contact variability. Bioimpedance measurement presents another promising alternative. While it can be less obtrusive, its accuracy is generally lower than direct respiratory sensors, and sensitivity can be affected by electrode placement and movement artifacts. Ultimately, the choice of method depends on the balance between comfort and signal quality.

#### 3.1.5. Ballistographic Sensors

The potential of ballistographic (BCG) sensors for contactless sleep monitoring opens compelling avenues for tracking biosignals without directly applying sensors on the body. BCG operates effectively through integration into everyday objects like mattresses, bed frames, and chairs, enabling unobtrusive, long-term sleep assessment. This approach is beneficial for tracking HR, HRV, RR, and broader physiological signals that indicate sleep health and quality. It is effective in identifying a range of sleep disturbances including insomnia, sleep apnoea, bruxism, RLS, nocturnal epilepsy, sleepwalking, and narcolepsy [186]. However, BCG is generally less effective in capturing fine neural activity typical in EEG-based sleep stages, making it better suited for general monitoring and longitudinal studies.

A leading example of a BCG-based device is the Emfit QS Active (Emfit Ltd., Vaajakoski, Finland) sleep monitor, placed beneath the mattress, which continuously records HR, HRV, RR, sleep stages, movements, recovery, stress levels, snoring, and overall sleep quality. The band-pass-filtered BCG sensor signal is available as “get API”. The low band 0.07–3 Hz is sampled at 25 Hz. The high band is of 1–50 Hz and sampled at 100 Hz. Emfit sends data to its cloud, where they are used to improve evaluation algorithms and possible individualisation [187]. Mack et al. [188] employed two mattress pressure pads for BCG in a sleep-monitoring system to assess HR and RR in 40 healthy subjects, in conjunction with PSG. Their low band was set at 25–514 mHz and their high band at 785 mHz–18 Hz, and they implemented peak detection and Hilbert transform analysis. Zhao et al. [189] utilised oil pressure sensors embedded in a micromovement-sensitive mattress to assess sleep apnoea syndrome by applying a knowledge-based support vector machine (KSVM) model, processing HR and RR data from 42 subjects over three nights. Yi et al. [190] developed a non-invasive hydraulic bed sensor for sleep stage classification, comprising four small pressure sensors under the mattress that capture small-amplitude movements, including BCG signals during each cardiac cycle and respiratory phases. Using SVM and KNN (K-nearest neighbour) models, they achieved 85% accuracy with a kappa of 0.74 for REM, NREM, and wakefulness detection. Further studies that demonstrate BCG’s versatility include that of Silva et al. [191], who applied Murata SCA11H (Murata Electronics, Vantaa, Finland) BCG sensors with a random forest algorithm to classify sleep stages. Alivar et al. [192] described a BCG-based motion detection algorithm within a smart bed system that effectively quantified restlessness, with Neyman–Pearson and sequential detection methods achieving 95% and 96% sensitivity for sleep movement, respectively. Liu et al. [193] identified OSA by exploiting event phase segmentation of BCG signals, yielding a precision of 94.6% and recall of 93.1%, as validated against 3790 OSA events. Event segments are classified into OSA events or non-OSA events using a backpropagation neural network. Xian Li et al. [110] used a piezoelectric film sensor for BCG monitoring in 32 subjects, providing foundational data for future BCG-based vital sign monitoring. Wang et al. [194] applied BCG to assess the severity of sleep apnoea, estimating the apnoea–hypopnoea index by identifying sleep-related respiratory events. As their methodology, they utilised wavelet decomposition and a Physio_ICSS-based algorithm. In terms of clinical validation, Nurmi et al. [195] tested an accelerometer-based BCG sensor, validated with PSG in 20 subjects, showing parameter accuracy within a 95% confidence interval. Hwang et al. [196] established an accurate apnoeic events monitoring method using a polyvinylidene fluoride (PVDF) film. Their procedure was based on the extraction of the respiratory signal from PVDF data, principal component extraction and data segmentation, threshold determination, and apnoeic event decision. For min-by-min ratios, they classified sleep apnoea with a sensitivity of 72.9%, specificity of 90.6%, and accuracy of 85.5%. Another smart device is a MEMS 3D accelerometer and pressure-sensor-based belt by He et al. [197], which is placed under the patient and aims to detect vital signs, snoring events, and sleep stages. The accuracy of snoring detection is 97.2%, and that of sleep stage detection is 79.7%. The combination of BCG and actigraphy is also increasingly popular, as noted by Jaworski et al. [127,198], as it enhances movement and cardiovascular data interpretation for comprehensive sleep analysis. Another device, the Withings Sleep Analyzer (Withings, Issy-les-Moulineaux, France) [199], is a unique combination of two powerful sensors placed under the mattress at chest level with a one-time setup. A sound sensor identifies audio signals specific to snoring and cessation of breathing episodes, and a pneumatic sensor measures HR, RR, and body movements across the mattress. It allows in-depth analysis of sleep cycles and detection of sleep apnoea and its severity at a medical grade.

The narrative review by Balali et al. [200] provides a comprehensive overview of innovations in respiratory signal extraction, cardiorespiratory interactions, and AI applications in BCG monitoring outside clinical settings. They highlight the benefits of BCG in cost-effectively improving clinical and home sleep monitoring. Lastly, Sadek et al. [201] present an in-depth review of sensor technologies for BCG, detailing signal processing methods for analysing HR, RR, and sleep stage classification, demonstrating BCG’s expanding role in sleep health monitoring.

#### 3.1.6. Acoustic-Based Devices

Acoustic sensors represent a promising, non-contact approach to sleep monitoring, leveraging sound analysis to assess physiological and environmental factors without body-worn devices. Their capacity to detect and interpret signals such as breathing patterns, snoring, coughing, and ambient noise is invaluable for monitoring sleep-related breathing disorders like sleep apnoea, as well as disturbances like restless leg syndrome and sleep talking. Acoustic sensors effectively capture respiratory events, monitoring rate and rhythm changes without disrupting the sleeper’s natural environment. Some limitations exist, such as potential signal interference from environmental noise and variability in complex respiratory condition analysis. However, advancements in acoustic signal processing, machine learning, and noise-filtering algorithms are addressing these limitations, enhancing the reliability of acoustic sensing in identifying sleep stages and respiratory events. Some products and research use their own microphone designs, but a large portion relies on mobile phone microphones for practical reasons.

Romero et al. [202] used acoustic screening to detect OSA in 103 participants through deep neural networks, achieving sensitivities and specificities of 0.79 and 0.80, respectively, for moderate OSA, and 0.78 and 0.93 for severe OSA, making it suitable for implementation on consumer smartphones. Markandeya et al. [203] and Nakano et al. [204] further monitored sleep apnoea, with Nakano’s study emphasizing snoring as a critical sound indicator for sleep apnoea. Penzel et al. [205] employed tracheal sounds for sleep apnoea diagnosis with the PneaVox (CiDELEC, Sainte-Gemmes-sur-Loire, France) sensor, designed with an airtight plastic chamber to minimise ambient noise and capture tracheal sounds accurately. This sensor is placed near the suprasternal notch and attaches via double-sided adhesive tape. It records respiratory sounds typically in the 200–2000 Hz range and snoring sounds from 20 to 200 Hz. In a study conducted on 20 children, the PneaVox demonstrated high reliability compared to a traditional polygraph (PG) device, indicating its utility in paediatric apnoea identification. A wearable medical device, AcuPebble SA100 (Acurable Limited, London, UK) [206], also utilises acoustic sensing to detect OSA. This compact, circular device (2.9 cm diameter, 1.4 cm height, 7 g weight) is affixed to the neck with disposable medical adhesive. It uses piezoelectric MEMS microphones and algorithms to capture and analyse respiratory events, heart rate, and breathing rhythm throughout the night. Although it does not provide direct PPG data or absolute oxygen saturation levels, it can detect oxygen desaturations through acoustic signal features. The device achieves a high diagnostic accuracy for OSA, with a specificity of 96.8% and sensitivity of 92.7%. Rodriguez-Villegas et al. [111] developed a compact (3.74 × 2.4 × 2.1 cm, 17 g) wearable acoustic sensor for detecting apnoea and hypopnoea, with a small microphone chamber affixed to the neck via adhesive patches. The device demonstrated 77.1% sensitivity and 99.7% specificity in apnoea and hypopnoea detection. Fang et al. [207] developed a wireless acoustic sensor attached near the nose with a commercial headset and MEL frequency cepstrum analysis for recording respiratory data during sleep, while Werthammer et al. [208] focused on infant apnoea detection, comparing respiratory sounds to trans-thoracic impedance and ECG.

#### 3.1.7. Radar System Devices

Radar-based sensors enhance user comfort by eliminating the need for wearable devices or physical contact. They are installed at a distance, such as on a ceiling or bedside table, ensuring minimal intrusion while maintaining accurate respiratory monitoring. This makes them especially suitable for long-term sleep studies and for populations sensitive to traditional sensor-based setups, such as children or elderly individuals.

Resuli et al. [209] developed a non-invasive device for monitoring respiration and sleeping posture, using a radio frequency (RF) sensor. The researchers used the Vayyar RF (Vayyar, Yehud-Monosson, Israel) with a carrier frequency of 6.014 GHz to collect signals for 13 different sleeping postures. All reflections were captured by a frequency-modulated continuous wave (FMCW) signal. The collected data were compared with a respiration belt. The RF sensor was placed on the ceiling, 2.3 m above the bed. The results showed 90% accuracy for RR estimation with the chest facing directly toward the sensor, 87% with the head positioned on the opposite side of the bed, and 86% while sitting. Turppa et al. [210] used another FMCW radar sensor for measuring RR, HR, and HRV during sleep. The study involved ten subjects in different lying positions. The fast Fourier transform (FFT)-based cepstral analysis was used for HR extraction, and the autocorrelation function was applied to the phase signal for RR extraction. The carrier frequency of the radar was 24 GHz with a 250 MHz bandwidth. The measurement system achieved a correlation of 86% for HR and 91% for RR, when compared with reference signals acquired by the certified PSG device, Embla Titanium (Raftopoulos, Athens, Greece). A very interesting device is Somnofy (Vitalthings AS, Trondheim, Norway) [112], which, in the form of an alarm clock, uses radar to detect RR, sleep phases, and restlessness, while also monitoring habits, lighting, atmospheric pressure, air quality, humidity, and temperature. Somnofy is an impulse-radio ultrawideband radar with a carrier frequency of 23.8 GHz. For signal processing, it uses FFT every second for each 20 s time window of measured data. In the study by Toften et al. [211], they used this device for measuring RR during sleep of 37 healthy adult subjects. Another six healthy participants were recruited for a 3-month-long use of the Somnofy device during sleep in a home environment. The results of the study showed Bland–Altman 95% limits of agreement ranging from −0.07 to −0.04 respirations per minute, compared with a reference RIP sensor. Further analysis showed that measurements were more accurate during deep sleep (NREM 3) and light sleep (NREM 1 or 2) than during other sleep stages (wake and REM). Dong et al. [212] designed a custom radar-based system with an algorithm for identifying respiratory variables and extracting respiratory phases and amplitude during sleep. The system consists of a radar sensor with a 24 GHz signal, a microcontroller unit (MCU) for signal preprocessing, and a Wi-Fi module for transmitting data to the cloud server. The system uses a zero-crossing time (ZCT) motion removal algorithm combined with locally estimated scatterplot smoothing (LOESS) for constructing respiratory waveforms from radar data. LOESS, which applies local regression to smooth the data, effectively removes the heartbeat component from the mixed chest movement signal. The measured data from the radar system were compared with data from the gold-standard PSG, simultaneously measured for ten subjects. Experimental results revealed an accuracy of 97% for respiration-to-respiration interval (RRI), 93% for inhale duration, and 92% for exhale duration assessment. Based on the accurate detection of RRI, it was also possible to distinguish between REM and NREM sleep. The SleepScore Max (SleepScore Labs, Carlsbad, CA, USA) [213] is a non-contact sleep monitoring device designed for bedside use, eliminating the need for wearable sensors. It measures movement, breathing, and environmental factors such as light and temperature to assess sleep quality and duration. Results are presented as a personalised SleepScore™, accessible via a companion app that offers evidence-based guidance for improving sleep. Validated in over a dozen peer-reviewed studies, it provides some of the most accurate non-contact sleep tracking outside clinical settings. Studies also demonstrate its ability to enhance sleep quality within one week of use. The study [214] presents a non-contact sleep monitoring device called S+. It operates by emitting low-power radio wave pulses at a frequency of 10.5 GHz to detect body movements. The effective range of the device is 1.5 m, ensuring accurate measurement of the intended person. The device is designed to detect respiratory patterns, overall body activity, room temperature, light, and sounds. It evaluates sleep stages—light sleep, deep sleep, REM, and wakefulness. The device’s accuracy for sleep–wake detection was 87%, compared to PSG. Its sleep sensitivity, exceeding 90%, was notably higher than its specificity, which ranged from 70% to 75%. The accuracy in evaluating individual sleep stages reached 68% for each stage.

The last device, WiFi-Sleep [215], does not directly fall under radar devices, but it still works on the principle of influencing the RF signal by human physiology, so we will include it here. WiFi-Sleep is designed for practical application in real-life environments, offering a reliable solution for long-term sleep monitoring. This innovative system tracks sleep across four stages through the key components of data collection, detection of respiration and body movements, and sleep stage classification. By utilizing standard Wi-Fi devices, WiFi-Sleep delivers a non-intrusive, cost-effective, and real-time method for comprehensive sleep analysis. The system operates with a pair of Wi-Fi transceivers, strategically placed with the subject positioned between them. WiFi-Sleep uses a combined CNN and bidirectional long short-term memory (BiLSTM) network to classify sleep stages. A multiscale CNN extracts features from sliding windows that match the duration of sleep stage episodes, while the BiLSTM integrates transitional information across the entire night. Future developments will focus on expanding the system’s ability to detect sleep-related conditions like chronic insomnia, RLS, and sleep apnoea. Additionally, the system’s functionality will be enhanced by refining the analysis of respiration waveforms, tracking body movements, and detecting PLMS, leading to improved accuracy.

Further research on radar-based sleep monitoring is extensively discussed in the following review articles [216,217,218].

Radar-based sensors are a modern alternative for respiratory and sleep monitoring, providing a non-contact solution that eliminates the discomfort associated with wearable devices. These sensors can measure key physiological parameters, including RR, HR, and body movement, making them ideal for long-term and at-home sleep monitoring. Their accuracy depends on positioning, distance, and signal processing techniques, but recent advancements in ML have improved their performance. While they offer high user comfort and minimal intrusion, challenges remain, such as sensitivity to the external interference, motion artifacts, and higher costs compared to traditional wearable sensors. Despite these limitations, radar-based systems are becoming a valuable option for sleep and respiratory monitoring, offering a balance between convenience and reliable data acquisition.

#### 3.1.8. Breath Gas Monitoring Devices

Temperature, humidity, and pressure sensors are suitable for sleep monitoring, and they can analyse breath during sleep and be useful for detecting early physiological changes [219,220,221]. The use of these sensors is based on the fact that exhaled air is warmer and more humid and contains more CO_2_ compared to inhaled air. Although most of the exhaled air is nitrogen, oxygen, water, and carbon dioxide, even a low concentration of volatile organic compounds can provide valuable information about various diseases, which also include neurodegenerative disorders [222,223]. The advantages of the temperature, humidity, and pressure sensors are non-invasiveness, painlessness, and the possibility of long-term monitoring. Recently, sensors made of flexible materials that can be adapted and seamlessly integrated into face masks or attached as a nasal patch have been preferred and developed [224,225,226].

Inhalation and exhalation generate airflow that mechanically interacts with pressure sensors, and the signals are further transformed into measurable electrical signals. An interesting innovation is that of self-powered breath sensors that simultaneously sense and harvest energy by utilizing the piezoelectric or triboelectric effect [227,228]. Temperature sensors commonly use thermistor materials such as the patch temperature sensor [229], nanofiber membrane colorimetry with thermochromic dye [230], or pyroelectric nanogenerators [227,231,232]. Exhaled breath temperature can effectively indicate inflammatory markers and changes in bronchial blood flow, making it a useful tool for identifying respiratory conditions such as asthma or lung cancer [233]. Cao et al. [234] proposed a wearable respiratory sensor based on thermally sensitive materials to monitor normal and abnormal breathing, ideal for sleep monitoring. Pang et al. [235] fabricated a smart face mask capable of recognizing eight breathing patterns. The sensor uses novel 3D carbon nanofiber mats as active materials to simultaneously realise pressure and temperature sensing. The moisture content of inhaled and exhaled air is a very important indicator of the physiological state of the monitored person. Humidity sensors monitor the interaction between the sensing material and water vapor in the gas based on the amount of water molecules on the surface of the material where they bind. Monitoring changes in relative humidity records the frequency and intensity of breathing and distinguishes nasal and oral breathing [236]. These changes indicate respiratory health, sleep quality, hypertension, etc. Honda et al. [113] developed a highly stable humidity sensor that can wirelessly monitor sleep apnoea in real time and in a home environment. The flexible humidity sensor is on a mask and has a ZnIn_2_S_4_ nanolayer, which is sensitive to humidity, with high sensitivity and stability for more than 150 h. Ma et al. [237] designed a low-cost, flexible, and easy-to-process paper moisture sensor for monitoring sleep breathing in the form of a patch. They achieved high sensitivity and application for monitoring sleep apnoea. The sleep mask NiteAura (Linkface, New York, NY, USA) for breathing care monitors breathing conditions and breathing during sleep, helping people with sleep-disordered breathing and setting appropriate conditions to help achieve deeper sleep [238]. It has multiple built-in sensors—for humidity, temperature, and IMU.

There are several articles about humidity, pressure, and temperature sensors, and for use in monitoring early stages of diseases and determining the overall physiological condition of the patient, their incorporation into wearable systems with an overall evaluation of multiple factors is suitable.

A comprehensive comparison of all the mentioned basic devices, along with their key features, is summarised in Table 4.

**Table 4:** Technical details of basic sleep monitoring devices.

Type	Application	Sensing Element	Key Parameters	Ref.
PPG	HR, SpO_2_, sleep stage	PPG (2× LED + PD), temperature, 3D IMU in ring	[Oura Ring] 106 subjects, ML using five-fold cross-validation, Sleep/wake accuracy 94% from accelerometric model and 96% from ANS and circadian features, Four-stage detection 57% and resp. 79%, ARM Cortex MCU, Bluetooth	[138]
PPG	HR, SpO_2_, sleep stage, movement	PPG, accelerometer, gyroscope on wristband	[Xiaomi Band 9] Ambient light sensor, Bluetooth, 45 subjects, Sleep stage accuracy 78%, Sensitivity 89%, Specificity 35%, κ = 0.22	[126]
PPG	HR, SpO_2_, sleep stage, movement, ECG, OSA	PPG, IMU, temperature in smartwatch	[Apple watch] Temperature, Ambient light sensor, Bluetooth, Processor S10 SiP, Memory 64 GB, GPS, Sleep stage agreement 53% (κ ^1^ = 0.2), Sensitivity 50.5–86.1%, Precision 72.7–87.8%	[129,142,146]
PPG	HR, SpO_2_, movement, OSA, ODI	PPG, accelerometer in ring	[O_2_ Ring] 190 subjects, ODI sensitivity 87.30% and specificity 78.70%, SVM model for OSA with sensitivity 97% and specificity 50%, Sampling rate 150 Hz, BLE, Recording time 16 h, HR accuracy ±2 bpm, SpO_2_ accuracy ±3%	[130,145]
PPG	HR, SpO_2_, RR, HRV, skin temperature, stress, sleep stages	PPG (3× green + red + IR LED, 4× PD)	[WHOOP 4.0] Sleep stage agreement 65% (κ = 0.52)	[146,147]
PPG	HR, SpO_2_, ECG, EDA, sleep patterns, stress, OSA, movement	PPG, BT, 3D accelerometer in smartwatch	[Fitbit Sense 2] NFC, Ambient light sensor, Wi-fi, GPS + GLONASS, Bluetooth, OSA sensitivity 88%, OSA specificity 52%, TST and SE overestimation 10%, Sleep stage sensitivity 61.7–78%, Precision 72.8–73.2%	[125,148]
PPG	HR, SpO_2_, RR, sleep stages, central and obstructive apnoea/hypopnoea	3D accelerometer and PPG in glove	[UpNEA] MAX-30101 PPG, MAX-21105 IMU, PPG sampling rate 100 Hz, Accelerometer sampling rate 50 Hz, BLE, Tachycardia/bradycardia/atrial fibrillation/premature ventricular contraction detection, Accuracy 75.1% for apnoea/hypopnoea detection, Central vs. obstructive accuracy about 83.2%	[124]
PPG	HR, SpO_2_, RR, OSA	In-ear PPG	16-bit, MSP430F1611 microcontroller	[149]
PPG	HR, SpO_2_, RR, head position, apnoea	PPG (red + IR LED), 3D accelerometer on the nasal septum	[MORFEA] MAX-30102 PPG, LSM6DSM accelerometer, Sampling rate 50 Hz, Modulation of PPG by breath, PSD ^2^ and PWA ^3^ method, Sensitivity 89% and precision 93% of apnoea detection, Bluetooth, Recording time 9 h	[150]
Actigraphic	Movements, light exposure	3D MEMS accelerometer, light sensor	[MotionWatch 8] Sleep patterns, PLMS detection, Circadian rhythm disorders, Memory 4 MBits, Recording time 3 months, Weight 9.1 g without strap	[155]
Actigraphic	Movements, ambient and body temperature, light	2D accelerometer, RGB—IR light, temperature	[ActTrust 1] Sleep patterns and activity, Circadian rhythm disorders, Memory 4 MB, Recording time 3 months, Weight 38 g	[156]
Actigraphic	Movements, ambient and body temperature, light exposure	3D accelerometer, RGB—IR light, temperature	[ActTrust 2] Sleep patterns and activity, Circadian rhythm disorders, Accelerometer sampling rate 25 Hz, Memory 8 MB, Resolution 12-Bit, Digital time display, Recording time 3 months, Weight 35 g	[156]
Actigraphic	Movements, ambient and body temperature, light exposure	3D accelerometer, RGB—IR light, temperature, melanopic light, off-wrist capacitive sensor	[Act Lumus] Sleep patterns and activity, Circadian rhythm disorders, Accelerometer sampling rate 25 Hz, Memory 8 MB, Resolution 12-Bit, Bluetooth, Recording time 1 month, Weight 31 g	[157]
Actigraphic	Movements, light exposure	Accelerometer, light sensor	[ActiGraph wGT3X-BT] Sleep patterns and physical activity, Weight 19 g, Sampling rate 30–100 Hz, Memory 4 GB, Recording time 25 days, BLE	[158]
Actigraphic	Movements, OSA	IMU, temperature	[SleepActa] RTC, Sampling rate 100 Hz, 78 subjects, Dormi algorithms (Waso, TST, SE, SRI ^4^), CE Class I medical device, MCC ^5^ 0.4 for mild AHI and MCC 0.3 for severe AHI	[107,160]
Actigraphic	HR, activity	3D accelerometer, PPG	[Somno-Art] Sleep classification, Insomnia, OSA, Narcolepsy detection, AI algorithms for automatic sleep analysis, Bluetooth, Accelerometer sampling rate 250 Hz, Sleep/wake accuracy 87.8%, Sleep stages accuracy 68.5%, Recording time 40 h	[162]
EEG	EEG, HR, breathing, body movement and position	Four-channel EEG, PPG, 3D IMU, respiration in headband	[Muse S] Sleep tracking and evaluation	[166]
EEG	EEG, HR, SpO_2_, movement, breathing temperature	Five-channel EEG, PPG, respiration, accelerometer on headband	[Dreem 3S] Sleep tracking, AI quality evaluation and disturbance diagnosis	[167]
EEG	EEG, HR, SpO_2_, CBT, body movement and position, snoring	Single-channel EEG, PPG, six-axis IMU sensor, sound, pressure sensor on the forehead	[UmindSleep] Sleep tracking, Forehead temperature, AI evaluation and disorder diagnosis	[108,169]
EEG	EEG, sleep tracking	Highly elastic memory foam, flexible electrodes	Sleep tracking	[170]
EEG	EEG, EOG, chin EMG	10× EEG, 1× EOG on the forehead, EMG electrodes	Sleep tracking	[171]
Respiratory	RR, tidal volume, minute ventilation, body position	Resistance-based sensor, IMU	Sleep disorder screening, Sleep staging, Respiratory pattern detection, Posture and activity detection, Bluetooth, Class IIa certified medical device	[109]
Respiratory	RR, breathing rhythm and depth	Textile RIP integrated into a suit, 3D accelerometer	Smart signal processing algorithm, Sampling rate 10 Hz, Wireless communication, Peak power consumption 140 mW, Radio transmission range 20 m	[175]
Respiratory	Respiratory effort, body position	IMU sensor	MCU CC2650 with ARM Cortex-M3, 16-bit resolution IMU, Wireless communication	[176]
Respiratory	Sleep apnoea	Strain gauge sensor	Sampling rate 10 Hz, Bluetooth, CNN ^6^, Accuracy 0.7609, Sensitivity of 0.78, Specificity of 0.72	[177]
Respiratory	RR, HR, HRV, ECG, skin temperature	ECG, thermometer, accelerometer	Adhesive chest patch, Single use and fully disposable, Sleep staging, Wireless communication, Class IIa certified medical device	[178]
Respiratory	RR, RE, HR, SpO_2_, sleep apnoea	PPG, ECG, SCG	PPG sampling rate 200 Hz, ECG sampling rate 120 Hz, SCG sampling rate 500 Hz, Sleep staging, Bluetooth, FFNN ^7^, RCNN ^8^, Recording 10 h, Sensitivity 100%, Precision 95%	[179]
Respiratory	Sleep apnoea	BioZ ^9^ sensor	BioZ sampling rate 1024 Hz, ECG sampling rate 512 Hz, Stimulation signal 8–160 kHz, Accuracy 72.8%, Sensitivity of 58.4%, Specificity of 76.2%	[185]
AFE ^10^	RR, ECG, EEG	ADS129xR	Eight channels, 24-bit, Sampling rate 250 Hz–32 kHz, CMRR ^11^ 115 dB, Internal oscillator	[180]
AFE	Respiration, ECG	AFE4960	Two channels, 22-bit, Single ADC, SPI and I^2^C interface, Sine wave or square wave excitation	[181]
AFE	Respiration, ECG, optical HR	AFE4500	Four input channels, 22-bit, single ADC, SPI and I^2^C interface	[182]
AFE	Respiration, ECG	ADAS1000	Five acquisition channels and one driven lead, SPI/QSPI interface, AC and DC lead-off detection	[183]
AFE	Respiration, ECG	MAX30001	High input impedance (>1 GΩ), SPI, 32-word ECG, Eight-word BioZ, FIFO ^12^, EMI ^13^ filtering, ESD ^14^ protection, DC lead-off detection	[184]
BCG	RR, HR, HRV, sleep, movement, snoring, stress	Dynamic ferro-electret under mattress	[Emfit QS] Sleep monitoring, Stress level, Sleep quality and classification, Low band 0.07–3 Hz at 25 Hz, High band 1–50 Hz at 100 Hz, cloud data	[187]
BCG	HR, RR	Two pressure pads on mattress	[NAPS] BCG evaluation in sleep, Overnight sleep studies of 40 subjects, Low band 25–514 mHz, High band 785 mHz–18 Hz, Sampling rate 150 Hz, 12-bit, Peak detection and Hilbert transform analysis	[188]
BCG	HR, RR	Set of oil pressure sensors in mattress	16-bit, Sampling rate 100 Hz, KSVM ^15^ model, 42 subjects/three nights, Apnoea precision rate 90.46% and recall rate 88.89%	[189]
BCG	HRV, RR variability	Hydraulic transducers under the mattress	Sleep quality and sleep-related disorders, SVM and KNN classification methods, Sampling rate 100 Hz, Sleep stage detection accuracy 85%, κ = 0.74	[190]
BCG	HR, HRV, RR, stroke volume	Murata SCA11H sensors (IMU) under mattress	Sleep management, Random forest algorithm, Sleep phase classification, Wi-Fi	[191]
BCG	HR, RR	300 × 580 mm electromechanical film sewn into a fitted sheet	Quantification of sleep quality, restlessness, Neyman–Pearson detection test, Sequential detection algorithm, 16-bit, Sampling rate 250 Hz, 94% and 95.2% accuracy in sleep and restlessness state identification	[192]
BCG	RR, OSA events	Micromovement sensor in mattress	Apnoea phase, Respiratory effort phase and arousal phase, 38 subjects, Backpropagation neural network, Accuracy 94.6%, Recall 93.1%	[193]
BCG	HR, RR, sleep quality	700 × 30 mm piezoelectric film sensor beneath the mattress	Sampling rate 140 Hz, 32 subjects, AMPD ^16^ algorithm, Correlation coefficient 0.95, MAE 1.78 bpm for HR, Correlation coefficient 0.98, MAE 0.25 rpm for RR	[110]
BCG	HR, RR, sleep apnoea syndrome	Four pressure sensors in mattress	Sleep apnoea syndrome severity, 136 subjects, Resolution 16-bit, Wavelet decomposition, Physio_ICSS-based algorithm, Accuracy 94.12%	[194]
BCG	HR, HRV, RR, RRV, respiratory depth, movement	Murata SCA11H sensors (IMU) under mattress	Sleep stage detection, 20 subjects, Sampling rate 1 kHz, Correlation coefficient 0.97 for HR, 0.67 for HF HRV, 0.54 for LF HRV, 0.54 for RR, 0.49 for RRV, Wi-Fi	[195]
BCG	HR, RR, apnoea and hypopnoea	4 × 1 array PVDF-film-based sensor under silicon pad on mattress	26 apnoea patients + 6 healthy subjects, NI-DAQ 6221 (National Instruments, Austin, TX, USA), Sampling rate 250 Hz, PCA ^17^ method, Correlation coefficient for AHI 0.94, Apnoea detection with 72.9% sensitivity, 90.6% specificity, and 85.5% accuracy	[196]
BCG	HR, RR, snoring, sleep stages classification	MEMS ISM330 DLC 3D accelerometer and pressure sensor array on mattress	STM32F411 ARM processor, FFT analysis, Accuracy for HR 1.5 bpm, for RR 0.7 rpm, Snoring recognition 97.2%, Sleep stage prediction 79.7%	[197]
BCG	Sleep stages, HR, HRV, RR	Murata SCA11H + Apple watch 8 + actigraphy device	Six subjects, Nonlinear methods, LSTM model, 73% agreement with PSG	[127]
BCG	HR, apnoea, snoring	Pneumatic and sound sensor under mattress	[Withings Sleep Analyzer] Medical-grade apnoea detection, Sleep cycle detection, Bluetooth, Wi-Fi	[199]
Acoustic	OSA, respiratory sounds, apnoea, AHI	Smartphone	DNN architecture, 3× CNN layers, Adam optimiser, Mel-frequency analysis, Sampling rate 256 Hz, 103 subjects, Sensitivity 0.79 and specificity 0.80 for moderate OSA, Sensitivity 0.78 and specificity 0.93 for severe OSA	[202]
Acoustic	OSA, respiratory sounds	Smartphone iPhone 7	Calibrated by oesophageal pressure manometry, ML algorithm, 13 subjects, Prediction of ΔPes ^18^ with MAE ^19^ 6.75 cm H_2_O, r = 0.83	[203]
Acoustic	OSA, snoring, apnoea, AHI	Smartphone	FFT analysis, 10 kHz Sampling rate, 50 subjects, Snoring time correlation r = 0.93, AHI correlation r = 0.94, OSA sensitivity 0.7, OSA specificity 0.94	[204]
Acoustic	OSA, snoring	Tracheal sound and suprasternal pressure sensor PneaVoX	Sensitivity 99.4%, Specificity 93.6%	[205]
Acoustic	Apnoea	Tracheal sensor AcuPebble SA100	63 subjects, OSA accuracy 89.77%, Central vs. obstructive apnoea accuracy 82.54%	[206]
Acoustic	Apnoea, hypopnoea	Tracheal sensor WADD	WADD and SOMNO automated software, 20 healthy and 10 apnoea-diagnosed subjects, Apnoea detection sensitivity 88.6% and specificity 99.6%	[111]
Acoustic	OSA, snoring	Wireless headset Plantronics M165 near nose	Sampling frequency 11 kHz, Mel-scale-based features, eight subjects, Snore detection accuracy 96.1%, Abnormal detection result accuracy 93.1%	[207]
Acoustic	Apnoea	Body-worn audio amplifier	MSP430 microcontroller, SPP, Orthogonal matching pursuit algorithm, Accuracy 80%, Bluetooth, Streaming 8 kb/s	[208]
Radar	RR, restless time	Vayyar FMCW ^20^ radar over bed	6.014 GHz, 14 transmitting and 13 receiving antennas, 13 different sleeping postures, Distance 2.3 m, RR accuracy 86–90%	[209]
Radar	HR, RR, sleep analysis	FMCW radar over bed	24 GHz, 250 MHz bandwidth, FFT based on cepstral and autocorrelation analyses, 11 subjects, HR correlation 86%, RR correlation 91%	[210]
Radar	RR, sleep stages, restlessness	Radar sleep monitor in form of alarm clock	[Somnofy] 23.8 GHz, Environment monitoring (sound, light, pressure, air quality, humidity, temperature), Night reports, Sleep assessment, Alerts	[112]
Radar	RR, sleep scoring	Radar sleep monitor in form of alarm clock	[Somnofy] 23.8 GHz, FFT, 37 subjects, RR with MAE 0.18, Accuracy of sleep detection 0.97, Accuracy of wakefulness detection 0.72	[211]
Radar	RR, inhale/exhale duration, NREM/REM stage detection	Radar-based IoT system on bedside wall	2 × 4 linearly polarised antenna array on PCB, Distance 40–100 cm, FIR filter (VMD ^21^, CEEMDAN ^22^, LOESS ^23^ algorithm), AMPD algorithm, Sampling rate 100 Hz, RR accuracy 97%, Inhale duration accuracy 93%, Exhale duration accuracy 92%, Wi-Fi	[212]
Radar	RR, movement, sleep stage detection	Radar on bedside table	[SleepScore Max tracker] Automated sleep scoring	[213]
Radar	RR, movement, sleep stage detection	Radar on bedside table	[S+ ResMed] 10.5 GHz, Emitting power 1 mW, Distance 1.5 m, Environment monitoring (room temperature, light, and sounds), 27 subjects, Sleep detection accuracy 93.8%, Wakefulness detection accuracy 73.1%	[214]
Radar	Body movements, respiration, apnoea	Wi-Fi-based system	[WiFi-Sleep] Sleep stage analysis, CNN, BiLSTM ^24^ , Accuracy for sleep classification 81.8%, Future developments—detecting chronic insomnia	[215]
Breath gas	Sleep monitoring, apnoea, hypopnoea, breathing	Platinum thermal sensor on patch	Response time 0.07 s, Sensitivity 1.4‰ °C^−1^, Sampling rate 32 Hz, 16-bit resolution, 10th-order Butterworth 3 Hz low-pass filter, RR filter 0.2–0.5 Hz, Bluetooth	[234]
Breath gas	Sleep monitoring, respiration	Pressure, temperature sensors in facemask	Recognizing eight breath patterns, 3D carbon nanofiber mats, discrimination between oral and nasal breathing, human body’s physiology analysis	[235]
Breath gas	Respiration, apnoea, RR, NREM/REM stage detection	Humidity sensor in facemask	Highly stable, Real-time wireless monitoring of sleep apnoea, ZnIn_2_S_4_ nanolayer, High sensitivity and stability, Operating time 150 h	[113]
Breath gas	Respiration, apnoea, breathing	Easy-to-process paper humidity sensor on patch	Low-cost, Flexible, High sensitivity 5.45 kΩ/% RH, Repeatability 85.7%, Sampling rate 18 Hz, Battery 3.7 V	[237]
Breath gas	Respiration, sleep breathing patterns, humidity level, temperature	Humidity, temperature, accelerometer, barometer, gyroscope, IMU sensors in facemask	[NiteAura] Breathing conditions during sleep, Helps with sleep-disordered breathing and sets appropriate conditions to achieve deeper sleep	[238]

^1^ Cohen’s kappa, ^2^ power spectral density, ^3^ pulse wave amplitude, ^4^ sleep regularity index, ^5^ Matthews correlation coefficient, ^6^ convolutional neural network, ^7^ feedforward neural network, ^8^ residual convolutional neural network, ^9^ bioimpedance, ^10^ analog front-end, ^11^ common mode rejection ratio, ^12^ first in–first out, ^13^ electromagnetic interference, ^14^ electrostatic discharge protection, ^15^ knowledge-based support vector machine, ^16^ automatic multiscale-based peak detection, ^17^ principal component analysis, ^18^ peak-to-trough differences, ^19^ median of absolute error, ^20^ frequency modulated continuous wave, ^21^ variational modal decomposition, ^22^ complete ensemble empirical mode decomposition, ^23^ locally estimated scatterplot smoothing, ^24^ bidirectional long short-term memory.

### 3.2. Advanced Sleep Monitoring Devices

In the field of sleep medicine, accurate diagnosis and monitoring of sleep disorders is crucial for effective treatment. Modern monitoring devices (Table 5) detect a wide range of sleep disorders, not only in the hospital but also in the home environment. These devices vary in complexity, ranging from simpler limited respiratory polygraphs that focus mainly on respiratory parameters to modular devices offering greater diagnostic capability, as well as full PSG systems, which provide comprehensive sleep analysis.

#### 3.2.1. Limited Respiratory Polygraphy

Limited respiratory polygraphy is performed with a monitoring device that records at least four channels, including two respiratory channels (e.g., airflow, respiratory effort), SpO_2_, and HR or ECG [239]. This device is typically classified as a Type III device according to the American Academy of Sleep Medicine (AASM). It is a simpler form of full-night PSG, which usually does not record EEG, EOG, or EMG, meaning that it does not provide detailed information about sleep stages [240]. This makes PG useful for diagnosing sleep-related breathing disorders, allowing for the determination of their severity, positional dependence, and type (e.g., obstructive, central, or mixed). A device that does not normally include EEG determines the AHI using total recording time (TRT) or time in bed (TIB). Due to its simple setup, it is ideal for home monitoring. Its main advantages include portability, affordability, and fast result evaluation [241,242,243]. Modern respiratory PG devices support the connection of additional sensors, enabling expanded and more detailed monitoring of diagnostic parameters, as further described in the following section.

An example of a typical PG system is the portable monitoring device Samoa (Löwenstein Medical SE and Co., KG., Bad Ems, Germany) [244]. The PG device uses a nasal pressure cannula for detecting pressure differences during breathing, and a thermistor is utilised to monitor temperature changes associated with respiration. The respiratory effort of the thorax and abdomen is captured by pressure rubber pads integrated into the straps. Additionally, a finger sensor measures SpO_2_, PPG, and HR, and an internal light sensor continuously tracks the brightness of the sleeping environment. The device also includes an integrated acceleration sensor to detect body position and activity. The Samoa device features a built-in microphone for capturing breathing sounds and is powered by a rechargeable Li-ion battery.

Another device used to diagnose sleep breathing disorders is the PG device Alice NightOne (Philips Respironics, Murrysville, PA, USA). The configuration of this device includes data channels for respiration, such as a nasal pressure cannula and a thoracic respiratory belt based on RIP to monitor respiratory effort. Additionally, it records snoring, SpO_2_, HR, PPG, and body position [245]. It can identify five different positions (upright, right side, left side, supine, and prone). The device is equipped with 4 GB of internal storage memory. To power the monitoring device, two AA alkaline batteries (1.5 V) or two AA rechargeable batteries (1.2 V) with a minimum capacity of 2400 mAh are required. The compact diagnostic device Alice NightOne measures 10.34 × 2.51 × 6.78 cm and weighs 84 g, excluding batteries and sensors. The sampling rate of the pulse oximetry is 62.5 Hz [246].

A wide variety of PG devices are available on the market from various leading companies in sleep medicine. In addition to those mentioned above, other devices include ApneaLink^TM^ Air (ResMed, San Diego, CA, USA) [247], Cadwell’s ApneaTrak (Cadwell Industries Inc., Kennewick, WA, USA) [248], SOMNOtouch RESP eco (SOMNOmedics AG, Randersacker, Germany) [249,250], and others. These devices vary in design, technical specifications, and price.

#### 3.2.2. Modular Systems

Modular monitoring systems are flexible diagnostic devices designed to allow clinicians and researchers to modify the configuration of recording parameters based on specific medical and research needs. These systems include removable sensors that allow targeted data collection while reducing the overall burden on the user. For example, in addition to the basic parameters that conventional PG can detect, modular devices can measure EEG, EOG, EMG of the chin muscles, or ECG by integrating additional sensors into the system for more comprehensive sleep analysis. By integrating these additional channels, modular systems bridge the gap between simpler respiratory polygraphs and full polysomnography setups, offering a scalable solution for more comprehensive sleep monitoring. The adaptability of modular systems extends their applicability to diverse patient populations, including paediatric and geriatric groups. Modular systems enhance operational efficiency by allowing healthcare professionals to upgrade devices incrementally rather than investing in entirely new systems. This approach reduces costs and increases accessibility in clinical and home settings. Some devices may prioritise portability and ease of use, while others focus on advanced monitoring capabilities or the possibility of real-time monitoring.

Such modular systems, like the SOMNOtouch^TM^ RESP (SOMNOmedics AG, Randersacker, Germany), offer versatility and straightforward operation thanks to their compact design and integrated features. The device is a modern touch-screen respiratory PG with a colour display and a built-in Li-ion battery. Besides the basic channels (airflow, effort, HR, SpO_2_), the SOMNOtouch^TM^ RESP includes an integrated actigraphy sensor for tracking movements. Based on body position and actigraphy, a detailed evaluation of sleep and wakefulness can be performed. The device can be upgraded to a PSG system by connecting EEG, EOG, EMG, and ECG channels. It is one of the lightest devices in its category, weighing only 64 g without sensors, and measuring 8.4 cm × 5.5 cm. The device employs an innovative algorithm to determine blood pressure using the pulse transit time (PTT) parameter [114,251].

Another device that is important to mention is the Nox T3s^TM^ (Nox Medical, Alpharetta, GA, USA). The polygraph is an advanced device for sleep monitoring that operates without the need for traditional EEG, EOG, or EMG signals, which are commonly used to track brain activity during different sleep stages. Instead, it uses a proprietary algorithm that identifies physiological changes that correspond to brain activity changes. This is done through Nox RIP technology and actigraphy. In addition to the basic monitored parameters, the device also features two integrated bipolar ExG channels that can record additional data, depending on the application. This portable system contains built-in 3D sensors for tracking body position and activity, as well as an upgraded microphone for clearer snoring detection. It offers 4 GB storage capacity and can record continuously for up to 24 h on a single AA battery. The device uses Bluetooth 5.0 LE, which allows it to measure signals from other compatible auxiliary devices. It also features a USB-C port under the battery cover for device configuration and data download. The Nox T3s^TM^ device is configured with Noxturnal 6.0 PC software, which also enables reviewing, organizing, analysing, and summarizing all the signals captured by the device. The device’s design makes it easy to use in both clinical and home settings [174,252].

The portable device Embletta^®^ MPR (Natus, Middleton, WI, USA) is a respiratory PG with exceptional functionality that can be configured into a PSG online wireless recorder by adding modules. With a basic module, the device can record additional channels: two EEG, one ECG, two EOG, and two EMG of lower limbs. With an advanced module, the device is upgraded to a full PSG, supporting six EEG channels, three chin EMG, two EMG of lower limbs, two EOG, and one ECG [253].

#### 3.2.3. Wireless PSG Devices

Traditional PSG examinations using digital PSG systems are performed in sleep laboratories, where medical professionals closely monitor the entire process. These PSG systems [7] provide accurate results and enable extensive analysis of physiological signals. However, their design places less emphasis on patient comfort. Traditional PSG systems rely on fully wired connections, which restrict patient movement during sleep, and recording units are typically larger and not portable [254]. Data transfer is conducted via physical connections to computer software, and there is no possibility of wireless data transmission via Bluetooth or Wi-Fi. On the other hand, cable connection provides reliable and stable data transmission.

The transition from wired to wireless PSG systems represents a breakthrough in patient convenience and usability. Key advantages include improved mobility, easier setup, and reduced interference from tangled wires. These systems are particularly suited for long-term studies requiring continuous monitoring over several nights [255]. However, the reliability of data transmission and potential interference in wireless environments remain challenges to address.

Modern PSG devices combine wired data transmission with wireless transmission from sensors, improving the movement flexibility of patients during sleep by reducing the number of cables. These PSG systems utilise wireless data transfer to local computers or cloud-based storage via communication protocols such as Bluetooth or Wi-Fi, making PSG systems portable and suitable for use in the home environment [256]. Such advances are in line with the goals of companies like SOMNOmedics, which focus on developing innovative solutions for sleep medicine. SOMNOmedics offers a wide range of equipment, from actigraphy devices and polygraphs to modular systems and full laboratory PSG, which are discussed in detail in the relevant sections of this article. A significant portable wireless PSG system by SOMNOmedics is the SOMNO HD [115,251]. It is unique for its wireless EMG sensors for the lower limbs, designed for PLMS detection. These wireless sensors for PLMS offer synchrony with precision of less than 60 ms and a sampling rate ranging from 64 to 512 Hz. The SOMNO HD supports a configuration of up to 55 channels, integrating both wireless and wired sensors to maximise versatility. It is designed to work with a maximum of six wireless sensors, enabling the collection of a broader range of physiological data while minimizing patient discomfort.

Another compact PSG system that integrates wireless technology is the Nox A1s^TM^ by Nox Medical. The device features a wireless pulse oximeter, an integrated snoring sensor, and a built-in accelerometer for measuring activity and body position. EEG electrodes are connected to a single cable leading to the device, which helps reduce the negative impact of the large number of cables on the patient’s well-being. The Nox A1s^TM^ (Nox Medical, Alpharetta, GA, USA) is equipped with a rechargeable battery that lasts for multiple nights of use, making it practical for home sleep studies [257,258].

#### 3.2.4. Wearable Devices

The evolution of advanced wearable devices has significantly transformed sleep monitoring by leveraging breakthroughs in sensor miniaturisation, algorithm development, and device ergonomics. Early wearable systems often fell short of matching the precision of traditional PSG conducted in clinical settings. However, innovations such as compact sensor arrays, extended battery life, and streamlined designs have enhanced both user convenience and functionality. Modern wearable PSG devices now integrate multi-channel sensors capable of monitoring key physiological parameters, including EEG, EOG, EMG, and vital signs such as HR and respiratory patterns, enabling a comprehensive analysis of sleep health [117,259]. Reliability of such home-based PSG is further enhanced through remote monitoring by enabling real-time adjustments and reducing failure rates [260]. Portable computerised PSG systems have also been validated against lab-based devices, demonstrating comparable signal quality, good agreement in sleep variables, and accuracy in diagnosing conditions like OSA [261].

Ongoing advancements in materials science are making adhesive patch systems popular and now replacing traditional bulky headgear. Systems with sensors integrated directly into the patches minimise sleep disruption. Developments in signal processing are increasing the accuracy of these devices and bringing wearable PSG technology closer to the reliability of traditional clinical-grade systems. Among wearable PSG systems, the patch-based Onera Sleep Test System (Onera Technologies B.V., Palaiseau, France) stands out for its innovative design and comprehensive monitoring functions, setting the standard in modern sleep diagnostics. This wearable wireless PSG system consists of four disposable patches placed on the forehead, thoracic and abdominal areas, and lower leg. The patch on the forehead records EEG, EOG, EMG, and SpO_2_, while the chest patch monitors ECG, activity, respiratory effort, sound pressure, and body position. A nasal flow pressure cannula is connected to the abdomen patch. The lower leg sensor detects leg movements [262]. The device is designed for user-friendliness, with an average hook-up time of approximately 4.5 min, which is more than a 77% reduction compared to traditional PSG montage [263]. The sensitivity, accuracy, and specificity for all sleep stages are high, except for NREM 1, which shows low sensitivity. The results from the Onera device highly correlate with traditional PSG [264]. The medical device company Compumedics introduced the Somfit^®^ system, which includes the Somfit device and an adhesive electrode. The device integrates neurological signals (single-channel EEG, single-channel EMG, and two channels of EOG) with channels designed for pulse arterial tonometry (PAT), which are commonly utilised in monitoring devices for OSA. The system measures parameters such as HRV, pulse, SpO_2_, and additional relevant metrics. The sleep staging algorithm of Somfit utilises deep learning, specifically built on a CNN architecture. Compumedics also developed the advanced Somfit^®^ Pro (Compumedics Limited, Abbotsford, Australia) system. In addition, this contains the Respifit module, which enables monitoring of additional parameters, like airflow, RR, body position, single-channel ECG, and thoracic effort via inductive belt [265]. Somfit’s automatic hypnograms and PSG showed an overall agreement of 76.14% across all sleep stages [266]. Another patch sensor with comprehensive analysis is presented by Kwon et al. [116]. One patch on the forehead is used for measuring EEG and EOG, and the second on the chin for measuring EMG. Electronic components are stored in elastic polyurethane, which makes them more comfortable to wear. The bottom side of the patches uses adhesive silicone with nanomembrane electrodes for better contact with the skin. Sleep stage tracking and diagnosis evaluation are done by CNN. Afterwards, the data are sent via Bluetooth to an external device. OSA detection accuracy reached 88.5%. In addition to the mentioned physiological parameters, this patch-based system incorporates sensors to monitor SpO_2_, carbon dioxide, and movement, enhancing the accuracy of apnoea detection. A study by Reis Carneiro et al. [267] presents a wireless solution for monitoring various electrophysiological potentials and detecting respiration signals, usable for several days without being affected by physical daily activities or bathing. The core material of the electrodes is a biphasic liquid metal composite, which provides a signal of high quality. The comfort of such a device lies in its reduced size and lightweight design. Thanks to digital printing, these bio-stickers can be easily tailored to specific research needs. This technology enables the creation of various configurations for monitoring a wide range of electrophysiological parameters, enhancing their flexibility and applicability in different settings.

An alternative wearable sleep monitor is the Sleep Profiler™ [268]. This wireless sleep monitor is designed to capture important parameters for assessing sleep quality. It is also capable of recording ECG, pulse, and snoring, and determining head movement and position. This device, in the form of a headband, provides detailed information about the sleep cycles, offering an accurate and efficient alternative to traditional polysomnography [269]. The advanced version of this sleep monitor, the Sleep Profiler PSG2™ (Advanced Brain Monitoring, Carlsbad, CA, USA), includes a wireless pulse oximeter, attachable chest and abdominal RIP belts, and a nasal flow cannula, providing a total of 13 channels for comprehensive monitoring. Xin Li et al. [117] present the WPSG-I wearable device consisting of a headband, pulse oximeter, and data processing software. The device monitors EEG, chin EMG, and EOG, which are extracted from frontal EEG electrodes. The headband of this device contains a 3D accelerometer for head position detection and an audio sensor for snoring and ambient sound. A wrist-mounted pulse oximeter measures SpO_2_ and wirelessly transmits the data to the headband’s recorder. Automated sleep staging has good accuracy compared to PSG, verified in healthy people and patients with neurological disorders. Other headbands for monitoring sleep, concentration, and physiological parameters, such as the FRENZ Brainband (Earable Vietnam Co., Ltd., Hanoi, Vietnam) [270,271], may also be of interest. The device is placed around the forehead and back of the skull, and uses a PPG sensor to capture SpO_2_ and HR. It also contains additional sensors for EEG, EMG, EOG, as well as a gyroscope and accelerometer, and uses the power of AI. All this can be viewed by users from a smartphone app. The device offers a solution in helping users to fall asleep more quickly and accurate monitoring of sleep status, and achieves high social acceptance through reliable assessment [272]. The market offers a wide range of devices for sleep monitoring and related disorders, many of which are suitable for home use. The choice of device depends on the specific preferences and needs of the user or healthcare provider. A comprehensive comparison of all the mentioned advanced devices, along with their key features, is summarised in Table 5. These wearable monitoring devices prioritise ease of setup, patient comfort, and reliable data collection. In general, wearable sleep monitors designed for home use should avoid complex setups that increase the risk of user error, while ensuring the quality of the collected data.

**Table 5:** Technical details of advanced sleep monitoring devices.

Type	Application	Sensing Element	Key Parameters	Ref.
PG	Flow, snoring, SpO_2_, HR, activity, light, thoracic and abdominal effort, body position	Pressure, thermistor, light, SpO_2_ sensor, accelerometer, chest/abdominal pressure pad, microphone	[Samoa and SleepDoc Porti^®^ 9] Bluetooth, Recording time 100 h, Weight 135 g, Battery 3.6 V	[244,273]
PG	Flow, thoracic effort, snoring, SpO_2_, HR, body position, PAP ^1^	Pressure, RIP ^2^, SpO_2_ sensor, accelerometer	[Alice NightOne] Sleep–wake determination, Memory 4 GB, Weight 84 g without battery and sensors, Bluetooth	[245]
PG	Flow, thoracic effort, snoring, SpO_2_, HR	Pressure, RIP, SpO_2_ sensor	[ApneaLink^TM^ Air] Memory 15 MB, Recording time 8 h, Weight 66 g	[247]
PG	Flow, thoracic and abdominal effort, snoring, SpO_2_, HR, body position	Pressure, thermistor, RIP, SpO_2_ sensor, microphone, accelerometer	[ApneaTrak Legacy] USB connection, Recording time 24 h, Weight 143.5 g	[248]
PG	Flow, thoracic effort, snoring, SpO_2_, HR, PPG, body position, activity, PAP	Pressure, RIP, SpO_2_ sensor, accelerometer	[SOMNOtouch RESP eco] USB, Additional sensor for abdominal effort/bruxism, Analysis of Cheyne–Stokes	[249]
Modular PG	Flow, snoring, SpO_2_, HR, thoracic and abdominal effort, PPG wave, body position, movement, PAP, extension to include EEG, EOG, ECG, chin EMG and leg EMG	Accelerometer, thermistor, pressure, RIP, SpO_2_ sensor, EEG, EOG, EMG attachable electrodes	[SOMNOtouch^TM^ RESP] Scalable to PSG, Memory 512 MB, Sampling rate 4–512 Hz, Built-in chest effort sensor and sensor for body position, BP monitoring, Weight 64 g	[114,251]
Modular PG	Flow, snoring, SpO_2_, HR, thoracic and abdominal effort, PPG wave, body position, activity, PAP, ExG ^3^	Pressure sensor, RIP, SpO_2_ sensor, accelerometer, microphone, two bipolar attachable ExG electrodes	[Nox T3s^TM^] Scalable to PSG, BLE, Memory 4 GB, Recording time 24 h, BodySleep technology by Nox, Weight 86 g	[174,252]
Modular PG	Flow, snoring and sound, SpO_2_, HR, activity, thoracic and abdominal effort, PPG wave, body position, extension to include EEG, EOG, ECG, chin EMG and leg EMG, ExG	Pressure, RIP, SpO_2_ sensor, accelerometer, 1× bipolar ExG electrodes, microphone, EEG, EOG, EMG attachable electrodes	[Embletta^®^ MPR] Scalable to PSG, Sampling rate 8 kHz, Resolution 24-bit, Recording time 24 h, Attachable ST/ST+ proxy for PSG Weight 153 g	[253]
Wireless PSG	EEG, EOG, ECG, chin EMG, flow, SpO_2_, HR, PPG wave, thoracic and abdominal effort, snoring, movement, body position, leg EMG, PAP, ambient light	Electrodes for EEG, EOG, chin EMG, EMG of limbs, and ECG, thermistor, pressure, RIP, SpO_2_, light sensor, microphone, accelerometer	[SOMNO HD] Up to 70 channels, Sampling rate up to 4 kHz/channel, Bluetooth real-time data transmission, Six wireless sensors available, Normal recording time 20 h, Online recording 12 h, Weight 190 g	[115,251]
Wireless PSG	EEG, EOG, ECG, chin EMG, flow, thoracic and abdominal effort, sound, SpO_2_, HR, PPG wave, body position, activity, leg EMG, PAP	Electrodes for EEG, EOG, chin EMG, EMG of limbs, and ECG, thermistor, RIP, pressure, SpO_2_ sensor, 3D accelerometer, microphone	[Nox A1s^TM^] Memory 4 GB, Recording time 30 h, Wireless PPG, Integrated snoring sensor, Built-in accelerometer, BLE ^4^, Ergonomic cable design, Weight 120 g	[257,258]
Wearable patch-based PSG	EOG, EEG, chin EMG, ECG, forehead SpO_2_, snoring, leg movements, airflow, respiratory effort, position, activity	EEG, EOG, ECG, EMG, bioimpedance, pressure sensor, sound, 3D accelerometer	[Onera STS], Sleep stage classification, Sleep-disordered breathing, PLMS, PSG with 15 channels, One-night acquisition	[262]
Wearable patch-based	EEG, EOG, EMG, HRV, HR, SpO_2_, snoring, head position, movement, ambient light, PAT	Three frontal electrodes, 3D accelerometer, PPG, microphone, two-channel EEG	[Somfit] Sleep stage classification, OSA, Insomnia and circadian rhythm disorders, AHI ^5^ and ODI ^6^, DL ^7^ on a CNN ^8^ architecture, EEG (24-Bit, 0.5–30 Hz), Agreement of 76.14% across all sleep stages, 7 days of recording, BLE	[265]
Wearable patch-based PSG	EEG, EOG, EMG, ECG, HRV, HR, SpO_2_, movement, ambient light, PAT, airflow, effort, position, RR, snoring	Three frontal electrodes, PPG, 3D accelerometer, microphone, inductive belts, nasal pressure cannula	[Somfit Pro] Sleep stage classification, Agreement of 76.14%, 2× EEG (24-Bit, 0.5–30 Hz), Breathing disorders, DL on CNN, Recording time 8 h, BLE	[265]
Wearable patch-based	EEG, EOG, chin EMG, SpO_2_, CO_2_ monitoring, movement	Nanomembrane electrodes	Sleep stage tracking, OSA detection, Evaluation by CNN, Bluetooth, OSA detection accuracy 88.5%	[116]
Wearable patch-based	EEG, EOG, EMG, ECG, respiration	Biphasic liquid metal composite electrodes	Sleep stage classification, Bruxism, Customizable digital printed bio-stickers, Light and flexible design	[267]
Wearable headband	EEG, EOG, EMG, ECG, HR, head position and movement, snoring	3× frontal electrodes, optical sensor, microphone, accelerometer	[SleepProfiler] Optional ECG and EMG electrodes, Memory 8 GB, Recording time 30 h, Eight channels, Bluetooth, 3.7 V battery 650 mAh, Weight 71 g	[268]
Wearable headband PSG	EEG, EOG, EMG, ECG, SpO_2_, HR, head position and movement, snoring, thoracic and abdominal effort	3× frontal electrodes, RIP, PPG, optical sensor, microphone, accelerometer, nasal pressure cannula	[SleepProfiler PSG2] Optional ECG and EMG electrodes, Recording time 26 h, Bluetooth, 3.7 V battery 650 mAh	[274]
Wearable headband PSG	EEG, EMG, EOG, SpO_2_, HR, head position, snoring and ambient sound	Frontopolar EEG and chin EMG electrodes, PPG, 3D accelerometer, audio sensor	[WPSG-I] Automated sleep staging with good accuracy compared to PSG	[117]
Wearable headband	EEG, EMG, EOG, SpO_2_, HR, breathing rhythm, head motion	Gold-plated brass electrodes and dry-sensing electrodes, PPG, accelerometer, gyroscope	[FRENZ Brainband] Determining sleep and concentration, AI, Accuracy of automatic sleep scoring 88%, Real-time, Supporting sleep quality with sounds	[270,271]

^1^ Positive airway pressure, ^2^ respiratory inductive plethysmography, ^3^ biopotential, ^4^ Bluetooth low energy, ^5^ apnoea–hypopnea index, ^6^ oxygen desaturation index, ^7^ deep learning, ^8^ convolution neural network.

Each of the mentioned systems has its own specific characteristics that influence its clinical application, availability, and diagnostic accuracy. Polygraphy devices, which record basic physiological parameters, have a more limited role in clinical practice, as they do not provide the detailed sleep stage analysis that full PSG systems offer. They are primarily used for detecting sleep-related breathing disorders, particularly obstructive sleep apnoea. Their lower cost and simpler application make them accessible not only to healthcare providers but also to patients for home-based sleep monitoring. On the other hand, modular PG systems offer greater flexibility in expanding monitored parameters. When needed, they can be enhanced with additional sensors, allowing them to function similarly to a full PSG system. However, expanding a basic PG setup to the level of a PSG system significantly increases the price of the device. The limiting factor is that most modular PG devices support a maximum of four EEG channels, which restricts the comprehensive EEG monitoring required for detailed sleep analysis in certain cases. Modular systems upgraded to PSG typically provide weaker technical parameters compared to wireless PSG systems. They often feature lower sampling rates, which can limit the precision and detail of EEG signal analysis.

Conventional PSG systems are still widely used in clinical settings, but due to their lack of portability and patient discomfort, they are gradually being replaced by modern wireless alternatives. Their robustness makes conducting examinations in a home environment particularly challenging. Traditional PSG relies on proper cable connections to ensure high-quality signal transmission. The cables must remain intact and undamaged, while the electrodes must maintain optimal impedance values to minimise noise and ensure reliable recording quality. Modern wireless PSG systems combine wired connections with wireless sensors and depend on reliable data transmission between the sensors and the receiver, with proper receiver placement being crucial for maintaining signal quality. However, despite their advantages, these systems come with certain challenges. In addition to their high cost, they face potential signal interference in environments with multiple sources of electromagnetic radiation. Their higher power consumption demands robust battery performance, which may limit the duration of monitoring. Furthermore, data security remains a critical issue, as the wireless transmission of sensitive medical information requires advanced encryption protocols to prevent unauthorised access.

Wearable compact advanced devices, which are approaching the capabilities of PSG systems, face challenges related to signal accuracy, limited options for adding sensors, as their configuration is usually fixed, and reduced channel availability compared to full PSG setups. Their smaller size and lightweight design can sometimes lead to compromised data quality. These devices are often designed with single- or two-channel EEG used for sleep assessment, lacking the ability for more comprehensive EEG analysis. Additionally, EEG-based wearable devices, such as those using headbands or headsets, still play a crucial role in ensuring patient comfort.

Nevertheless, technological progress continues to drive the development of increasingly sophisticated PSG systems with more compact designs, fewer cables, greater reliability, and improved user accessibility. These advancements contribute to more efficient and convenient sleep monitoring, making it more accessible for both clinical and home-based applications while maintaining high diagnostic accuracy.

### 3.3. Application in Neurological Disorders

Detection of sleep disorders is increasingly recognised as an early indicator of various neurological disorders, including Alzheimer’s and Parkinson’s disease, and may be crucial for early intervention and treatment. Devices that monitor changes during sleep are becoming increasingly important for early detection and treatment and are undergoing continuous development and improvement. They offer non-invasive methods to assess sleep patterns and identify anomalies associated with various conditions. We focused on clinical studies conducted in neurological patients, with a focus on specific devices. Most studies use classic PSG performed in hospital sleep departments, but recently, more research has begun to emerge showing the benefits of home PSG and smaller sensory devices.

Home-based sensors that can detect disease are of great interest, as they are inexpensive and easy to use, and can analyse early events in neurodegenerative diseases. For example, researchers are developing a headphone-like electroencephalography (EEG) device designed to monitor brain activity during sleep, suitable for early screening of Alzheimer’s and Parkinson’s disease by detecting specific EEG patterns associated with these conditions. The device is also capable of measuring other sleep-related variables (SpO_2_, body temperature, HR, and RR). However, clinical trials have not yet been conducted [275].

A study by You et al. [276] found that sleep dysfunction and β-amyloid (Aβ) deposition are associated, and that this deposition increases the number of nighttime awakenings, which also negatively contributes to memory impairment. β-amyloid deposition in the brainstem was in turn associated with daytime sleepiness. Although the study was conducted in elderly patients with disorders such as dementia and mild cognitive impairment, it shows that even the beginnings of amyloid protein deposition could lead to sleep disorders and thus alert to early events leading to neurodegeneration. In Alzheimer’s, a study using overnight PSG has also been published, finding that delayed onset of REM sleep is associated with higher levels of amyloid and tau proteins, which are markers of Alzheimer’s disease [277].

In PD, even more sleep disturbances can be seen in patients at an early stage, and this study therefore highlights the importance of monitoring early changes in neurodegenerative diseases. In their study, Dodet et al. [278] used overnight video-PSG in the Sleep Disorders Unit. The results showed that REM sleep latency was longer in PD patients, the percentage of N1 tended to be higher, and the percentage of REM sleep tended to be lower than in controls. Postuma et al. [279] found that idiopathic REM sleep disorder (iRBD) is a significant early indicator of neurodegenerative diseases, leading to a high conversion rate (approximately 6% of patients with iRBD convert to neurodegenerative disease each year, with more than 73.5% converting within 12 years), based on a study of sleep disorders using PSG.

Specifically, we have not yet found many home PSG devices that have been used in research for neurological disorders, although some of the above-mentioned devices meet the necessary parameters. For example, PSG devices such as the SOMNOwatch eco from SOMNOmedics are capable of being used for the diagnosis and management of PD, as they detect PLM/RLS movements and monitor tremor frequency and intensity. The SOMNOwatch eco is a compact advanced actigraphy device designed to objectively monitor sleep and movement patterns over several weeks. It is equipped with a 3D acceleration sensor that tracks body position and activity, as well as a light sensor to measure the ambient light level in the environment. It provides key parameters such as time in bed (TIB), sleep–wake analysis, circadian rhythm monitoring, 24 h sleep patterns, and a raster display for detailed information [280].

Another suitable device is the Dreem Headband by Beacon Biosignals [167], offering PSG quality in a lightweight and easy-to-use apparatus that includes five EEG sensors, a bone conduction speaker for audio output, and an accelerometer to measure movements, head position, and RR during sleep. The device is comfortable, efficient, and suitable for both home use and clinical studies, with EEG signal quality akin to PSG [281]. It uses deep learning and advanced digital signal processing to create algorithms that can predict EEG events more accurately and reliably than any expert. In addition to classic sleep analysis, AI quantifies sleep microarchitecture to search for new biomarkers of disease. Van den Bulcke et al. [282] performed a study with the device on mild to moderate AD patients and demonstrated a significant mean increase in SWS in response to targeted acoustic stimuli in those patients. Alternatively, Gonzales et al. [283] conducted a study in PD patients and demonstrated the suitability of the device for monitoring patients with neurodegenerative diseases and found that longer PD duration and rapid eye movements were associated with greater alertness and worse motor symptoms correlated with less deep sleep.

A similar device capable of detecting neurodegenerative diseases based on sleep biomarkers is the Sleep Profiler™ [268] that we mentioned above. This device is a wireless, lightweight, PSG sleep monitor with sensors for recording EEG, EOG, and EMG, which are necessary to characterise sleep time by individual stages. It also records ECG, pulse, head position, head movement, and quantitative snoring and is fully capable of detecting sleep abnormalities in AD and PD patients [284,285]. Similarly, other PSG systems are suitable for use in the detection of neurological diseases, as they meet all the parameters necessary for early diagnosis, even if they have not yet been used for these specific purposes. For instance, while the Nox A1s PSG device has not been tested with PD or AD patients, its features offer all the tools for early diagnosis of AD/PD [174,286]. Sleep disturbances are not only symptoms of advanced neurodegenerative diseases, but are also critical early indicators that home PSG, with its ability to assess sleep architecture in detail, should be able to detect, thus aiding in early diagnosis. These studies underscore the importance of PSG in identifying potential progression to neurodegenerative diseases, emphasizing the need for early detection and intervention. By enabling early detection of diseases such as PD and AD, home PSG offers significant potential to improve patient outcomes and slow disease progression. The continued development of PSG technology, including the integration of AI and wearable systems, promises to bridge the gap between clinical diagnosis and daily patient care. For example, a study by researchers at the Massachusetts Institute of Technology [287,288] has developed an innovative, non-invasive method for monitoring sleep and detecting neurological disorders using a system that uses a wall-mounted radio frequency (RF) device to emit low-power radio waves into the surrounding environment. As these waves bounce off a person’s body, the device captures data on breathing and movements during sleep. The information obtained is then processed using advanced AI algorithms to assess sleep stages and identify potential markers of neurological conditions. Best of all, it is a contactless method that does not disturb the monitored individuals or restrict their sleep. As innovation advances, home PSG represents a transformative tool in the early detection and treatment of neurodegenerative diseases, reshaping the future of sleep monitoring and neurological health.

### 3.4. Advances in Algorithms

As wearable devices continue to advance, the integration of smart algorithms, particularly those using AI, has significantly enhanced their ability to process and interpret physiological signals. Many devices now offer more precise monitoring, early detection of health anomalies, and personalised interventions.

The integration of AI into wearable technology not only improves the accuracy of health monitoring but also enables personalised healthcare solutions. By continuously learning from vast datasets, these devices can adapt to individual user profiles, offering tailored feedback and interventions that surpass the capabilities of traditional rule-based systems.

The availability of publicly accessible datasets is crucial for advancing research in such devices, as they enable researchers to develop, validate, and benchmark algorithms, fostering innovation and ensuring reproducibility in scientific studies. Open data sharing promotes collaboration across institutions, accelerates the discovery of new insights, and facilitates the integration of diverse data sources, thereby enhancing the robustness of research findings. Moreover, access to well-curated datasets allows for the application of advanced analytical techniques, including artificial intelligence and machine learning, ultimately contributing to improved healthcare outcomes.

Several publicly available datasets support the development of AI-driven algorithms for wearable devices, providing high-quality physiological signal recordings for research and application. The Table 6 above presents a selection of well-known datasets relevant to sleep staging, ECG, EEG, and other biosignal analyses, highlighting their key features and potential applications in wearable health monitoring.

In wearable devices equipped with sensors such as PPG, PSG, EEG, and EOG, advanced algorithms, particularly those utilizing AI, play a crucial role in processing and interpreting physiological data. These algorithms can be categorised based on the specific AI methodologies they use.

For instance, convolutional neural networks (CNNs) excel at capturing spatial hierarchies in data, making them suitable for analysing physiological signals. In sleep monitoring applications, CNNs have been utilised to classify sleep stages by analysing EEG signals. By learning spatial features from the input data, CNNs can effectively distinguish between different sleep stages, contributing to more accurate sleep assessments [298]. Additionally, recurrent neural networks (RNNs), particularly long short-term memory (LSTM) networks, are designed to capture temporal dependencies in sequential data. This makes them suitable for analysing time-series physiological signals. For example, in the context of PPG signal analysis, RNNs have been employed to detect sleep apnoea syndrome by learning temporal patterns associated with the condition. The use of RNNs in this context allows for the modelling of temporal dynamics in physiological signals, enhancing the detection of sleep-related disorders [299]. Combining CNNs and RNNs leverages the strengths of both architectures, capturing spatial features and temporal dependencies simultaneously. In wearable devices, hybrid CNN–RNN models have been applied to PPG signal analysis for tasks such as sleep staging using aligned PSG signals. The CNN component extracts spatial features from the input data, while the RNN component captures temporal dynamics, resulting in improved performance in classifying sleep stages [300]. While AI-driven techniques have become increasingly popular, traditional signal processing methods remain widely used in physiological signal analysis. For example, Fourier transform and wavelet transform are frequently applied to EEG and ECG signals to analyse frequency-domain characteristics [301]. Additionally, rule-based algorithms and statistical models, such as hidden Markov models (HMMs) and linear discriminant analysis (LDA), are employed in sleep staging and cardiac anomaly detection [302,303]. These conventional approaches offer interpretability and lower computational requirements, making them suitable for real-time processing in wearable devices.

The integration of these AI methodologies into wearable devices has strong potential to enhance the analysis of physiological signals, leading to more accurate health monitoring and personalised healthcare solutions.

## 4. Discussion and Future Directions

The growing demand for personalised health solutions and the integration of sophisticated sensor technologies are driving sleep monitoring technology forward rapidly and have achieved significant progress over the past decade. However, many challenges and opportunities that could shape the future of the field remain. Each type of device has its own advantages, parameters, and limitations. Wearable devices such as smartwatches, rings, and bracelets have become the most popular form of sleep monitoring, largely due to their affordability and ease of use. These devices primarily rely on PPG-based sensors to measure HR and HRV and SpO_2_, as well as accelerometers to track activity. The advantages of such devices include their affordability, portability, and user-friendly design, making them suitable for long-term use. PPG sensors are useful for estimating sleep stage. However, their accuracy is affected by movement artifacts, skin tone, and ambient light interference. Although they are convenient and widely available, they do not directly measure neural activity or respiration, which limits their accuracy with respect to sleep stage. Therefore, there is currently a great effort to develop algorithms to calculate blood pressure, respiration, etc., but their accuracy is still limited. Battery life is generally moderate (1–7 days), and the price varies depending on additional features, typically ranging from €50 to €800. Actigraphy is, on the other hand, a proven and recognised method for sleep monitoring, which primarily detects movement patterns. Wrist-based actigraphy devices are commonly used in clinical and research settings due to their affordability, long battery life (weeks to months), and ability to track sleep–wake cycles over extended periods. However, they do not differentiate between sleep stages and may misinterpret wakefulness as sleep. Ankle-based actigraphy, on the other hand, can detect periodic limb movements (PLMS) and restless leg syndrome (RLS), making it one of the few non-invasive tools for these disorders. These devices are inexpensive (€100–300) and require minimal maintenance. EEG headbands are among the most reliable wearable tools for identifying sleep stages, because they directly measure brain activity. These devices typically contain several dry electrodes placed on the forehead, sometimes along with EOG sensors that share the same electrode placement. Despite their high accuracy during sleep, their main limitation is user compliance. The need for continuous skin contact and potential discomfort during sleep can reduce long-term usability. Their battery life ranges from one to three nights, and they cost from €200 to €1500, with some requiring subscription-based analytics services. Athletes commonly use chest straps, which primarily measure RR but are now also suitable for HR and HRV. The focus is shifting from classic chest straps to more advanced ones based on changes in chest impedance. While chest straps offer better accuracy compared to wrist-worn PPG devices, they are not optimised for sleep and can be uncomfortable for extended overnight use. Battery life ranges from days to weeks, and costs range from €80 to €250. Respiratory sensors built into masks or nasal patches analyse exhaled gases, airflow, and humidity changes. These systems provide precise breathing data, making them valuable for studying sleep-disordered breathing. However, they are intrusive and less practical for everyday home use. Battery life varies, and costs range from €200 to €800.

Remote systems, such as BCG sensors, radar devices, and acoustic monitors, offer a non-intrusive alternative to wearable devices, and therefore, they offer the highest comfort. These systems are particularly advantageous for populations for whom wearing devices is impractical, such as the elderly or individuals with sensory sensitivities. Radar-based systems, for example, can capture detailed respiratory and movement data without physical contact, making them ideal for home environments. However, their adoption is hindered by higher cost, installation complexity, and susceptibility to environmental interference. BCG sensors, usually built into bed pads, are very convenient but can suffer from signal interference, for example when multiple people share a bed. Their accuracy in distinguishing sleep stages remains lower. Most models are long-lasting, require minimal maintenance, and range in price from €200 to €1500. Acoustic sensors detect breathing patterns, snoring, and other respiratory events associated with sleep disorders such as OSA. While they offer a convenient, non-contact alternative, background noise and room acoustics can affect accuracy. These systems generally lack the ability to provide detailed analysis of sleep architecture. The cost is relatively low. A user can literally start from €0 using a smartphone microphone. A stand-alone device costs €50–500, and maintenance is minimal. Radar sensors use radio frequency waves to detect micro-movements related to breathing and heart rate and can track sleep with minimal disruption to the user. However, their effectiveness decreases with increasing distance, and their placement must be optimised for reliable signal acquisition. Battery-powered models can last for weeks to months, while plug-in versions require continuous power. Prices range from €150 to €500.

Recent innovations have also led to the development of advanced systems that integrate multiple physiological parameters, including EEG, respiratory signals, and body temperature, to approach PSG-like accuracy. These systems, such as modular or wireless PSG devices, are particularly promising for clinical research and home diagnostics. Although these devices offer excellent accuracy, their higher cost, bulkier design, and technical complexity may limit widespread adoption. For example, limited respiratory polygraphy (PG) includes multiple physiological sensors (e.g., nasal cannula, chest belts, oximetry) for diagnosing sleep apnoea and respiratory disorders. These devices provide more comprehensive data than standard wearable trackers but require proper sensor placement and calibration, limiting their accessibility for non-expert users. Costs typically range from €1000 to €4000. We were able to obtain the prices of a few specific products; for example, Resmed ApneaLink Air™ [247] costs €1700, and SOMNOtouch™ RESP eco [249] costs around €3400. The price may vary depending on the choice of accessories. Modular PSG systems allow selective sensor combinations to balance comfort and data quality. While they provide gold-standard sleep measurements, they remain expensive and complex to set up, making them less practical for widespread home use. Purchase costs can exceed $5000, with additional maintenance and calibration expenses over time. For example, the modular PG Nox T3s [304] starts at €5900 and the SOMNOtouch RESP [114] at €5000. With EEG electrodes and online scanning, the price climbs to €14,000. Wireless PSG is the best but generally also the most expensive system. For example, NoX A1 [286] costs €18,000, and SOMNO HD [115] is estimated at €20,000. For a specific order of three SOMNO HDs with cameras, license, and evaluation PC, the price is €92,000. Regular stationary PSGs cost around €8000 in the case of Alice 6 LDxS [7] on average and €8500 for SOMNOscreen plus [305].

On the topic of specific prices, we can also mention prices in our own Slovak Republic. Listed prices for sleep examination start at €20 for night pulse oximetry, €90 for polygraphy, and €250–390 per person/night for PSG in a hospital. One complete PSG examination in a home environment increases to €1190, and this does not even account for more specific examinations. Within our project, a single PSG examination in a hospital for PD patients, including a neurological examination, patient questionnaires, etc., costs us €730.

For clarity, Table 7 provides a concise summary of the advantages and limitations of each technology.

Looking ahead, the integration of multimodal systems that combine multiple physiological measurements appears to be the most promising direction for sleep monitoring technology. By leveraging data fusion techniques, ML algorithms, and AI, these systems can achieve higher accuracy and greater diagnostic value [306,307,308,309]. In addition, the development of wearable devices specifically tailored for neurological disorders such as PD underscores the growing emphasis on personalised and specific monitoring solutions.

We are also actively involved in this area within the project “NAP—Twin on a Chip Brains for Monitoring Individual Sleep Habits” [310]. This project represents a new paradigm in science and technology, which aims to evaluate the potential of next-generation brain organoids, which would serve as miniature, personalised sleep models and predict early symptoms of PD. The project includes the transfer of sleep patterns from ePPG [10] and Dormi [107] devices to organoids, as well as the development of new algorithms for home PSG and the investigation of sleep habits of PD patients. We plan to provide a detailed description of this work in later publications.

The challenge for future research lies in striking a balance among accuracy, usability, user comfort, and cost. While advanced systems are approaching the capabilities of PSG, they must also address barriers to widespread adoption, such as those of convenience, affordability, and privacy. A promising direction is the modularity of systems, which allows for customisation of device configurations based on specific diagnoses and individual needs. A flexible approach in which different sensors—such as EEG for sleep stage determination, PPG for cardiovascular monitoring, or acoustic sensors for respiratory assessment—can be integrated as needed would increase usability and diagnostic capabilities. To achieve this goal, ensuring interoperability between devices from different manufacturers is crucial, allowing for seamless integration across platforms. Standardisation of sensor outputs, data formats, communication protocols, and validation methods would help maintain consistency and reliability, foster innovation, and make home sleep monitoring more clinically valuable and accessible. Artificial intelligence (AI) is poised to further transform sleep monitoring by automating and improving data analysis. AI-based processing can significantly reduce the burden of manual assessment, allowing healthcare professionals to focus on critical cases while improving overall diagnostic efficiency. Personalised sleep monitoring represents a major step forward, because AI, or neural networks, can learn an individual’s unique physiological patterns rather than relying on generalised algorithms designed for the average person. This enables earlier detection of subtle physiological changes that may indicate emerging pathologies, improving preventive care. Similar approaches have already proven successful in personalised medicine based on ECG Holter monitors and are now well-suited for sleep monitoring. In addition, embedded neural networks within sensing devices enable real-time data processing and on-device screening, reducing the amount of data transmitted and extending battery life. These advances will make sleep monitoring not only more accurate and effective, but also more sustainable for long-term home use. Research into innovative materials for wearable electronics, such as those described by Yi et al. [311] or the self-powered electronics presented by Chen et al. [312], may also bring great progress.

Privacy concerns include data confidentiality, potential misuse of data, and the psychological impact on participants. Sleep monitoring devices collect highly sensitive physiological data which in a medical setting are protected under health privacy regulations such as HIPAA (in the U.S.) and GDPR (in Europe). However, ensuring secure storage and transmission of these data is critical, especially for home-based monitoring where devices may rely on cloud storage or Wi-Fi connections, increasing vulnerability to data breaches [313]. Sleep data can potentially be used for purposes beyond medical care, such as marketing or insurance risk assessment. Home-based PSG devices, especially consumer-grade wearables, may share data with third-party companies, raising concerns about commercial exploitation without explicit consent [314]. Knowing one’s sleep is being continuously monitored can lead to anxiety or changes in behaviour, potentially affecting sleep patterns and undermining a study’s accuracy. Ethical protocols should include steps to minimise participant discomfort and ensure that data collection reflects typical behaviour [313]. While home-based monitoring offers convenience, it shifts some responsibility to the participant, such as managing device setup and maintenance. This autonomy can be empowering but may also increase the likelihood of errors or noncompliance if adequate training and support are not provided. Addressing these concerns requires robust ethical frameworks, secure data handling protocols, and transparent communication among researchers, healthcare providers, and participants. It also calls for balancing technological advancements with the protection of individual rights and well-being.

A collaborative effort among engineers, clinicians, and researchers will be essential to drive innovations that meet these requirements and to expand the use of sleep monitoring technologies in clinical, home, and research settings [315].

Sleep research is advancing rapidly, and the transition to wearable electronics and home measurements is unstoppable. We have been able to describe only a small part of the current state. If we attempted in this article to focus on every facet of the subject, its length would unfortunately be unbearable. Even describing the algorithms or automatic scoring systems used in detail would take up entire separate articles. Therefore, we bring to the attention of readers interested in this issue the following excellent reviews. Birrer et al. [254] provided a comprehensive overview of the reliability of sleep evaluation using wearable electronics, and Tran et al. [316] reported on their use in OSA. Lujan et al. [313] described wearable multi-sensors for monitoring sleep and circadian rhythms while providing a glimpse into the history, present, and future. De Fazio et al. [317] focused on methodology and wearable electronics in sleep dysfunctions. Zambotti et al. [318] focused on the state of the science and recommendations for using wearable technology in sleep and circadian research. Kwon et al. [260], cited earlier, contributed recent advances in wearable sensors and portable electronics for sleep monitoring. Cay et al. [319] added AI methods, and Peake et al. [320] wrote a review of consumer wearables and mobile applications for monitoring stress and sleep.

## 5. Conclusions

In conclusion, sleep monitoring technologies are advancing rapidly, driven by innovations in wearable and remote devices. While these tools vary in design and functionality, they share a common goal of improving accessibility, accuracy, and relevance for both general and clinical applications. The future lies in integrating these technologies into multimodal, user-friendly systems capable of providing precise insight into sleep health and its links to broader physiological and neurological conditions in real-word settings. With continued interdisciplinary collaboration, sleep monitoring is poised to transform not only our understanding of sleep, but also its role in predicting and managing complex disorders.

## Figures and Tables

**Table 1:** Basic PSG parameters of adults and their physiological values [5].

Sleep Parameter	Duration ^1^
Sleep onset latency	≤30 min
NREM 1	3–5%
NREM 2	45–55%
NREM 3	10–20%
REM	20–25%
REM sleep latency	60–100 min
Wakefulness after sleep onset	1–5%
Sleep efficiency	>85%

^1^ In minutes or % of sleep duration.

**Table 2:** Classification of sleep-disordered breathing severity in adults [5,63].

Classification of Severity of Sleep-Related Breathing Disorders	AHI ^1^
Without sleep-related breathing disorders	<5
Mild severity	5 ≥ AHI < 15
Moderate severity	15 ≥ AHI < 30
Severe severity	≥30

^1^ Apnoea–hypopnoea index.

**Table 3:** Summarisation of significant physiological changes during individual sleep stages in healthy people [25,30,33,67,73].

	Awake	NREM 1	NREM 2	NREM 3	REM
**EEG**	In a relaxed wakeful state with closed eyes, EEG activity typically shows frequencies in the alpha wave range (8–13 Hz), with low amplitude. During non-relaxed wakefulness, beta waves (14–30 Hz) are commonly observed.	Characterised by LAMF ^1^ activity with predominant theta waves (4–7 Hz). Alpha activity dissipates, and typical vertex sharp waves lasting up to 0.5 s are visible.	Typical theta waves (4–7 Hz) with low to medium amplitude. Presence of sleep spindles (short bursts of 11–16 Hz) and K-complexes (sharp delta waves lasting 1 s), which play key roles in sleep maintenance and memory consolidation. Phase duration is about 25 min and lengthens with each cycle, comprising about 45% of TST ^2^.	Characterised by slow delta waves (0.5–3.5 Hz) with high amplitudes of at least 75 μV in frontal leads. Delta waves constitute more than 20% of the duration of an EEG epoch.	Desynchronised EEG activity with sawtooth waves of 2–4 Hz and moderate amplitude, appearing in small clusters in frontal leads. Dream activity occurs with an emotional undertone. REM sleep consolidates memory traces and strengthens memory.
**EOG**	Varies depending on activity.	Slow eye movements.	Minimal or absent eye activity.	Minimal or absent eye activity.	Rapid eye movements.
**EMG**	Sustained tonic activity with high amplitude.	Lower amplitude of tonic activity compared to wakefulness, but still high.	Lower than in NREM 1.	Lower than in NREM 2.	Muscle atonia, occasionally interrupted by brief muscle twitches.
**HR**	It depends on physical activity and emotional stimuli. The normal range is from 60–100 bpm.	Slight decrease compared to wakefulness.	Decrease of 5–8% compared to wakefulness.	Decrease of 5–8% compared to wakefulness.	Irregular.
**HRV**	Affected by age, stress, exercise, and sleep quality. Overall HRV is lower in older people compared to younger ones.	Overall HRV increases, LF component decreases, LF/HF ratio decreases, HF component increases.	Overall HRV increases, LF component decreases, LF/HF ratio decreases, HF component increases.	HRV peaks, LF components are at their lowest, LF/HF ratio decreases, HF components are at their highest.	Significant decrease in HRV, LF component increases, LF/HF ratio increases, HF component decreases.
**RR**	The normal range is between 12 and 20 breaths per minute in adults.	Slower rate compared to wakefulness, regular.	Slower rate compared to wakefulness, regular.	Slow and regular.	Rate equal to or higher than wakefulness, breathing becomes shallow.
**BP**	It is higher than during sleep, usually in the normal range around 120/80 mmHg.	Decrease compared to wakefulness, less pronounced than in NREM 2 and NREM 3.	Decrease of 5–14% compared to wakefulness.	More significant decrease than in NREM 2, 5–14% lower than wakefulness.	Increase of approximately 5% compared to NREM sleep.
**CBT**	It is higher than during sleep and is regulated by the circadian rhythm.	Decrease compared to wakefulness.	Decrease compared to wakefulness.	Largest temperature drop.	Increase compared to NREM sleep.

^1^ Low mixed frequency, ^2^ total sleep time.

**Table 6:** Publicly available datasets.

Dataset Name	Type	Basic Info	Size	Ref.
DEAP Dataset	EEG, ECG, PPG	Multimodal dataset for emotion analysis, including EEG, ECG, and PPG signals from 32 participants watching 40 one-minute music videos.	32 participants, 40 one-minute trials each	[289]
MIT-BIH Polysomnographic Database	PSG (EEG, ECG, respiration)	Contains over 80 h of polysomnographic recordings, each with an ECG signal annotated beat-by-beat, and EEG and respiration signals annotated with respect to sleep stages and apnoea.	18 records—over 80 h of PSG records	[290]
AnxiECG-PPG Database	ECG, PPG	Synchronised ECG and mobile-acquired PPG recordings from 47 healthy participants during baseline, physical activation, and psychological activation conditions.	47 participants	[291]
Sleep-EDF Database	PSG (EEG, EOG, EMG)	Contains whole-night polysomnographic sleep recordings, including EEG, EOG, and EMG signals, with corresponding hypnograms manually scored by trained technicians.	197 whole-night PSG recordings	[292]
ISRUC-Sleep Dataset	PSG (EEG, EOG, EMG)	Collected at the Hospital of Coimbra University, includes recordings from subjects with various sleep disorders, featuring EEG, EOG, and EMG signals.	118 subjects divided in three subgroups	[293]
CODE-15% Dataset	ECG	A dataset of 12-lead ECGs with annotations, containing 345,779 exams from 233,770 patients, obtained through stratified sampling from the CODE dataset.	345,779 exams	[294]
PTB-XL Dataset	ECG	Contains 21,837 clinical 12-lead ECGs of 10 s length with a sampling frequency of 500 Hz, including various annotations.	21,837 recordings	[295]
I-CARE Database	EEG, ECG	Includes baseline clinical information and continuous EEG and ECG recordings from comatose patients following cardiac arrest, admitted to intensive care units.	32,712 h of data in 80,809 recording segments from 607 patients	[296]
DREAMER Dataset	EEG, ECG	Provides EEG and ECG recordings, as well as emotion annotations in terms of valence and dominance of people watching film clips.	23 participants	[297]

**Table 7:** Comparative summary of different devices’ technologies.

Type	Main Measured Parameters	Advantages	Limitations	Battery Life	Cost Range
PPG-Based	HR, HRV, SpO_2_	Widely available, non-invasive, good for sleep trends	Affected by movement, limited sleep staging accuracy	1–7 days	€50–800
Actigraphy Wristbands	Motion–sleep–wake Patterns	Inexpensive, validated, long battery life	Cannot detect sleep stages, misinterprets wakefulness	Weeks to months	€100–300
EEG Headbands	EEG, EOG	High accuracy in sleep staging	Can be uncomfortable, requires proper placement	1–3 nights	€200–1500
Chest Straps	HR, HRV, RR	Good accuracy for HRV and respiration	Discomfort during sleep	Days to weeks	€80–250
BCG Sensors in Mattresses	HR, RR, Movement	Non-contact, comfortable	Susceptible to motion artifacts from bed partners	Years	€200–1500
Acoustic-Based Devices	Snoring, Apnoea Detection	Non-invasive, good for apnoea detection	Accuracy affected by background noise	Months	€50–500
Radar-Based Systems	RR, HR	Completely non-contact, low maintenance	Accuracy depends on placement and distance	Weeks to months	€150–500
Respiratory Mask Sensors	Airflow, Humidity, Gas Composition	Precise respiratory analysis	Intrusive	Variable	€200–800
Limited PG	Respiration, Oximetry	Good for sleep apnoea screening	Requires sensor setup and calibration	Variable	€1000–4000
Modular PSG Systems	EEG, EOG, EMG, Respiration, etc.	Comprehensive sleep analysis	Expensive, complex setup	Variable	€5000+
