# Correction: Vitazkova et al. Transforming Sleep Monitoring: Review of Wearable and Remote Devices Advancing Home Polysomnography and Their Role in Predicting Neurological Disorders. *Biosensors* 2025, *15*, 117

**DOI:** 10.3390/bios15080508

**Published:** 2025-08-06

**Authors:** Diana Vitazkova, Helena Kosnacova, Daniela Turonova, Erik Foltan, Martin Jagelka, Martin Berki, Michal Micjan, Ondrej Kokavec, Filip Gerhat, Erik Vavrinsky

**Affiliations:** 1 Institute of Electronics and Photonics, Faculty of Electrical Engineering and Information Technology, Slovak University of Technology, Ilkovicova 3, 81219 Bratislava, Slovakia; helena.kosnacova@stuba.sk (H.K.); erik.foltan@stuba.sk (E.F.); martin.jagelka@stuba.sk (M.J.); martin.berki@stuba.sk (M.B.); ondrej.kokavec@stuba.sk (O.K.); filip.gerhat@stuba.sk (F.G.); 2 Department of Psychology, Faculty of Arts, Comenius University, Gondova 2, 81102 Bratislava, Slovakia; daniela.moskalova@uniba.sk

**Text Correction**

There was an error in the original publication [1]. A correction has been made to Section 3.1. Basic Sleep Monitoring Devices, subsection 3.1.1. PPG-Based Devices, Paragraph 2, Line 19: New text:

A study by Svensson et al. [143] validated the accuracy of Oura’s Sleep Staging Algorithm 2.0, showing that its measurements closely matched PSG, with sleep staging accuracy ranging from 75.5% for light sleep to 90.6% for REM sleep. Attention should also be given to the related commentary by Lolli et al. [144], which highlighted minor inconsistencies.

**References**

As a result of the correction, reference 143 was inserted [2]. The original citation 143 by Lolli is now citation 144 [3]. With this correction, the order of some references has been adjusted accordingly. The authors state that the scientific conclusions are unaffected. This correction was approved by the Academic Editor. The original publication has also been updated.
